# Effect of Polyplex Size on Penetration into Tumor Spheroids

**DOI:** 10.1021/acs.molpharmaceut.3c00397

**Published:** 2023-10-09

**Authors:** Cristina Casadidio, Jet E. M. Hartman, Bárbara S. Mesquita, Ragna Haegebaert, Katrien Remaut, Myriam Neumann, Jaimie Hak, Roberta Censi, Piera Di Martino, Wim E. Hennink, Tina Vermonden

**Affiliations:** † Department of Pharmaceutical Sciences, Division of Pharmaceutics, Utrecht Institute for Pharmaceutical Sciences (UIPS), Utrecht University 99, 3508 TB Utrecht, The Netherlands; ‡ School of Pharmacy, Drug Delivery Division, University of Camerino, CHiP Research Center, Via Madonna delle Carceri, 62032 Camerino, Macerata, Italy; § Laboratory of General Biochemistry and Physical Pharmacy, Faculty of Pharmaceutical Sciences, Ghent University, 9000 Ghent, Belgium; ∥ Department of Pharmacy, “G. D’Annunzio” University of Chieti and Pescara, Via dei Vestini 1, 66100 Chieti, Chieti, Italy; ⊥ Recusol Srl, Via del Bastione 16, 62032 Camerino, Macerata, Italy

**Keywords:** gene delivery, 3D in vitro model, tumor penetration, tumor stroma

## Abstract

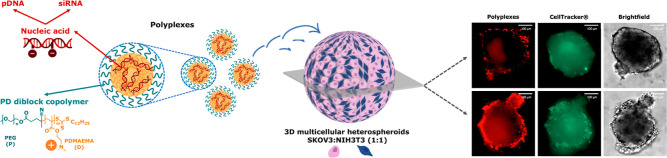

Ovarian cancer is one of the most lethal gynecological cancers in the world. In recent years, nucleic acid (NA)-based formulations have been shown to be promising treatments for ovarian cancer, including tumor nodules. However, gene therapy is not that far advanced in clinical reality due to unfavorable physicochemical properties of the NAs, such as high molecular weight, poor cellular uptake, rapid degradation by nucleases, etc. One of the strategies used to overcome these drawbacks is the complexation of anionic NAs via electrostatic interactions with cationic polymers, resulting in the formation of so-called polyplexes. In this work, the role of the size of pDNA and siRNA polyplexes on their penetration into ovarian-cancer-based tumor spheroids was investigated. For this, a methoxypoly(ethylene glycol) poly(2-(dimethylamino)ethyl methacrylate) (mPEG-pDMAEMA) diblock copolymer was synthesized as a polymeric carrier for NA binding and condensation with either plasmid DNA (pDNA) or short interfering RNA (siRNA). When prepared in HEPES buffer (10 mM, pH 7.4) at a nitrogen/phosphate (N/P) charge ratio of 5 and pDNA polyplexes were formed with a size of 162 ± 11 nm, while siRNA-based polyplexes displayed a size of 25 ± 2 nm. The polyplexes had a slightly positive zeta potential of +7–8 mV in the same buffer. SiRNA and pDNA polyplexes were tracked in vitro into tumor spheroids, resembling in vivo avascular ovarian tumor nodules. For this purpose, reproducible spheroids were obtained by coculturing ovarian carcinoma cells with primary mouse embryonic fibroblasts in different ratios (5:2, 1:1, and 2:5). Penetration studies revealed that after 24 h of incubation, siRNA polyplexes were able to penetrate deeper into the homospheroids (composed of only cancer cells) and heterospheroids (cancer cells cocultured with fibroblasts) compared to pDNA polyplexes which were mainly located in the rim. The penetration of the polyplexes was slowed when increasing the fraction of fibroblasts present in the spheroids. Furthermore, in the presence of serum siRNA polyplexes encoding for luciferase showed a high cellular uptake in 2D cells resulting in ∼50% silencing of luciferase expression. Taken together, these findings show that self-assembled small siRNA polyplexes have good potential as a platform to test ovarian tumor nodulus penetration..

## 1 Introduction

Ovarian cancer is the third most prevalent gynecological cancer throughout the world and is responsible for most deaths among these types of malignancies.^1^ Despite the relatively high number of available treatments, a promising and efficient cure warranting a high quality of life and patient survival is still lacking. However, with the emerging technology of gene therapy, this new treatment modality may become available to treat ovarian cancer. Among the wide range of available nucleic acid (NA)-based therapy strategies, one innovative approach is using short interfering RNA (siRNA), a double-stranded RNA that usually consists of ∼21 nucleotides, which blocks certain cellular pathways by silencing the gene of interest in the mRNA level and therefore inhibiting protein expression.^2,3^ However, gene therapy is not that far advanced in clinical applications due to instability of siRNA in vivo and low cell transfection efficiencies of naked nucleic acids upon in vivo administration.^4,5^ To overcome these challenges, nanocarriers that protect the siRNA and increase transfection efficiency are required, i.e., polymer, lipid-based systems, or viral vectors.^6−10^

One of the commonly investigated nanocarriers for nucleic acid delivery are cationic polymers that bind and condense the structure of negatively charged nucleotides to result in the formation of so-called polyplexes. These complexes are able to protect the NAs from enzymatic degradation and enable cellular uptake.^4,8,11−15^ A commonly used polymer to form these polyplexes is poly(2-(dimethylamino)ethyl methacrylate) (pDMAEMA), which displays cationic characteristics at physiological pH.^16,17^ By complexation of NAs with a block copolymer of the hydrophilic and noncharged poly(ethylene glycol) (PEG) and the cationic polymer, the resulting polyplexes have a shell–core structure, where the NA is condensed in the core and PEG is confined as a shell. PEGylation is commonly performed to shield the surface charge of polyplexes and to avoid/minimize aspecific cellular binding.^18−21^ In the present work, a mPEG-pDMAEMA diblock copolymer was used as a polymeric carrier for nucleic acid condensation and delivery.^18^

It has been shown that small polymeric micelles, i.e., 30–50 nm, are able to penetrate tumors better than bigger polymeric micelles (70–100 nm).^22,23^ It is, therefore, important to take the size of polyplexes into account as it will be relevant for in vivo studies regarding optimal delivery of nanoparticles and their penetration into tumor tissues. Indeed, looking more specifically at characteristics of ovarian cancer, at the severe stages of the disease, tumor nodules are present and widespread in the peritoneal cavity.^24−26^ To investigate the penetration of polyplexes into tumor nodules, spheroids that can mimic solid in vivo tumors can serve as a suitable model to investigate internalization of drug-loaded nanoparticles and polyplexes.^27−30^ Tumors do not only consist of cancer cells but also contain a variety of stroma cells that make up the complete tumor tissue, e.g., fibroblasts, macrophages, and endothelial cells.^29,31,32^ Cancer cells can transform fibroblasts into cancer-associated fibroblasts (CAFs), making these cells an important component of the tumor microenvironment (TME). CAFs are capable of remodeling the extracellular matrix (ECM) resulting in reduced penetration of nanoparticles.^32,33^

In this study, we developed a live-cell spheroids model as a fast-screening tool to investigate the penetration of polyplexes using confocal laser scanner microscopy (CLSM). To mimic in vivo ovarian ascites, homo- and heterospheroids of avascular ascites nodules were optimized by coculturing various types of ovarian cancer cells with primary mouse embryonic fibroblasts in different ratios. SiRNA and pDNA-based polyplexes were then tracked through the use of this platform and studied for their penetration ability to get insights of the effect of polyplex size and stroma on their penetration. Furthermore, to gain a deeper insight into the mechanism of nanoparticle penetration within the spheroids, uptake studies have been conducted on 2D mono- and coculture cells.

## 2 Materials and Methods

### 2.1 Materials

Unless indicated otherwise, chemicals were purchased from Sigma-Aldrich (Stenheim, Germany) and used as received. Prior to use, 2-(dimethylamino)ethyl methacrylate (DMAEMA) was passed through a column of alumina to remove the inhibitor prior to its polymerization. The pDNA pGL3-control reporter vector with simian virus 40 promoter (5256 bp) was obtained from Promega (Leiden, The Netherlands), amplified with DH5α competent *E. coli* bacteria cells, and purified using NucleoBond PC2000 DNA purification kit (Macherey-Nagel, Bioke, Leiden, The Netherlands). Dialysis tube membranes [molecular weight cutoffs (MWCO) of 3.5 and 10 kDa] were purchased from Fisher Scientific (Bleiswijk, The Netherlands). The siRNA that targets firefly luciferase and the negative control siRNA were provided by Horizon Discovery (PerkinElmer, UK) (Sequences in Table S1, Supporting Information). Midori Green was purchased from Nippon Genetics Europe (Düren, Germany). CellTracker Green CMFDA Dye, CellTracker Deep Red Dye, Wheat Germ Agglutinin-Alexa Fluor 488 conjugate, and 6× loading dye were provided by ThermoFischer Scientific (The Netherlands), whereas Hoechst 33342 was provided by molecular probes (Oregon, USA). Label IT Nucleic Acid Labeling Kit Cy5 was obtained from Mirus Bio LLC (WI, USA). The Luciferase assay kit and CellTiter 96 AQueous One Solution Cell Proliferation Assay (MTS) kit were purchased from Promega (Leiden, The Netherlands).

### 2.2 Synthesis of mPEG-p(DMAEMA) Block Copolymer

A diblock polymer methoxypoly(ethylene glycol) poly(2-(dimethylamino)ethyl methacrylate) (mPEG-pDMAEMA) was synthesized by reversible addition–fragmentation chain transfer (RAFT) polymerization, using a slightly modified method as previously reported.^34^ Briefly, DMAEMA (873 mg, 5.56 × 10^–3^ mol), commercially available mPEG_5000_-CTA (poly(ethylene glycol)-methyl ether (4-cyano-4-pentanoate dodecyl trithiocarbonate)) (100 mg, 1.85 × 10^–5^ mol), and azobis(isobutyronitrile) (AIBN, 3.70 × 10^–5^ mol) were dissolved in 3 mL of dry DMF, yielding a (DMAEMA)/(PEG-CTA)/(AIBN-Initiator) ratio of 300:1:0.2 equiv. Before starting the reaction, the mixture was degassed with a minimum of three freeze–pump–thaw cycles and after that the reaction mixture was placed in an oil bath at 70 °C and stirred for 8 h under a nitrogen atmosphere. The polymer solution, diluted with water, was then transferred into a dialysis bag (MWCO 14 kDa) and dialyzed against water for 2 days at 4 °C. The final mPEG–PDMAEMA (PD) block copolymer was recovered by freeze-drying, and the crude product was obtained as a white powder with a yield of 86%. The obtained polymer was characterized by ^1^H NMR spectroscopy and gel permeation chromatography (GPC) analysis.

#### 2.2.1 Kinetics of Polymerization

At predetermined time points, 300 μL samples of the polymerization mixture (composition see section above) were withdrawn and dialyzed against water for 1 day at 4 °C (MWCO 3.5 kDa) and subsequently freeze-dried to measure the degree of polymerization by ^1^H NMR and GPC. For ^1^H NMR measurements, samples were dissolved in dry CDCl_3_, and the percentage of conversion was calculated by comparing the integrals of DMAEMA signals at 4.1 ppm to the PEG signal at 3.8–3.5 ppm (as a reference). The remaining dried product was dissolved in DMF with 10 mM LiCl and its molecular weight and polymer dispersity index (*D̵*) were analyzed by GPC as described below.

### 2.3 Polymer Characterization

#### 2.3.1 ^1^H NMR Spectroscopy

The PEG macroinitiator and synthesized diblock copolymer were characterized with ^1^H NMR spectroscopy using an Agilent 400 MR-NMR spectrometer (Agilent Technologies, Santa Clara, CA, USA). Chemical shifts were referred to the residual solvent peak (δ = 7.26 ppm for CDCl_3_). Data analysis was performed using MestReNova Software (version 14.2.1).

#### 2.3.2 Gel Permeation Chromatography

The synthesized polymers were also characterized by GPC using a Waters Alliance System (Waters Corporation, Milford, MA, USA) equipped with a refractive index (RI) detector and a PLgel 5 μm MIXED-D column (Polymer Laboratories) using DMF containing 10 mM LiCl as an eluent. The column temperature was set to 65 °C and the flow rate was set to 1.0 mL/min, with a sample injection volume of 50 μL and a concentration of 5 mg/mL. Calibration was performed using PEG standards of narrow and defined molecular weights obtained from PSS (Germany). Data analysis was performed using Empower 3 Software (version 7.0).

### 2.4 Preparation of Polyplexes

Polyplexes were prepared using two types of cargo: pDNA and siRNA. For pDNA polyplexes, the PD polymer solution (concentration ranging from 57 to 517 μg/mL) and pDNA stock solution (100 μg/mL) were prepared in HEPES buffer (10 mM, pH 7.4). The siRNA polyplexes were prepared using siRNA and PD polymer stock solutions of 445 and 261–2610 μg/mL respectively, in the same HEPES buffer. Both pDNA and siRNA polyplexes were prepared with nitrogen/phosphate (N/P) molar ratios of 1, 5, and 10, by adding the polymer to the pDNA or siRNA solution. The resulting mixtures were vortexed for 10 s and incubated at room temperature for 20 min.

### 2.5 Cy5-Labeling of pDNA and siRNA

Stock solutions of pDNA and siRNA were labeled with Cy5 using a *Label* IT Nucleic Acid Labeling Kit Cy5, following the manufacturer’s protocol. More in detail, a ratio 1:1 (v/w) of NA (5 μL of 1 mg/mL NA stock solution) and Label IT Reagent has been used. After their incubation of 1 h at 37 °C, the samples were purified following the ethanol precipitation method according to the manufacturer’s protocol. The labeling density of pDNA-Cy5 (final base/dye ratio of 1:25 and labeling of 4%) and siRNA-Cy5 (final base/dye ratio of 1:20 and labeling of 5%) was determined with a microarray assay using a NanoDrop One UV–vis spectrophotometer (ThermoFisher Scientific, Isogen Life Science B.V., Utrecht, The Netherlands), using an extinction coefficient of 250,000 cm^–1^ M^–1^ of Cy5 at 650 nm. To obtain an equal fluorescence intensity of the polyplexes for the penetration studies, an adjusted concentration of Cy5-labeled NA per well was used. For this, the siRNA-Cy5 concentration was corrected by mixing labeled siRNA-Cy5 with nonlabeled siRNA (dilution 1:300), resulting in the same fluorescence intensity of pDNA-Cy5 per well.

### 2.6 Polyplex Characterization

#### 2.6.1 Dynamic Light Scattering

Hydrodynamic diameter and polydispersity index (PDI) of the formed polyplexes were determined with dynamic light scattering (DLS). Samples were prepared in HEPES buffer (10 mM), pH 7.4, as described in Section 2.2, with a final pDNA or siRNA concentration of 30 and 90 μg/mL, respectively. Measurements were carried out on a Zetasizer Nano S (Malvern Instruments, Malvern, UK) with an He–Ne laser of 633 nm and at 37 °C. Data were corrected for viscosity using the Malvern Zetasizer software (version 8.01).

#### 2.6.2 Laser Doppler Electrophoresis

The ζ-potential of the polyplexes was measured using laser Doppler electrophoresis on a Zetasizer Nano Z (Malvern Instruments) at 37 °C. Samples were prepared in HEPES buffer (10 mM, pH 7.4) with pDNA and siRNA final concentrations of 15 and 45 μg/mL, respectively.

#### 2.6.3 Fluorescence Correlation Spectroscopy and Loading Capacity

To confirm the hydrodynamic diameter of the Cy5-labeled siRNA polyplexes, fluorescence correlation spectroscopy (FCS) was utilized as previously described.^35^ Briefly, time traces of signal fluctuations were recorded when fluorescent particles diffused in and out of a confocal detection volume. Measurements were taken using a Nikon C1 confocal microscope (Nikon, Japan) outfitted with a photon counting instrument (PicoHarp 300, PicoQuant, Berlin, Germany), and a water immersion objection lens (60× Plan Apo VC, N.A. 1.2, Nikon) focused 50 μm above the glass bottom of the 96-well plate. SiRNA polyplexes were loaded in a 96-well plate, and time traces of 60 s were obtained of each sample in triplicate. The autocorrelation curves were analyzed with SymPhoTime (Picoquant) using a single-species triple-state model fit to determine the diffusion coefficient. The hydrodynamic radius (*R*_h_) of the polyplexes was determined using the Stokes–Einstein equation
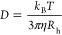
1where *D* is the diffusion coefficient, *k*_B_ is the Boltzmann’s constant, *T* is the absolute temperature, and η is the viscosity of the medium (HEPES 10 mM at pH 7.4). To determine the amount of siRNA per polyplex, we measured the amount of free siRNA before complexation. Then, we formulated them into polyplexes and measured the number of resulting polyplexes. An estimation of the amount of siRNA per polyplex then comes from the division of both numbers. As only the siRNA is fluorescently labeled, the free (nonlabeled) polymers are not interfering with the FCS measurements.^36,37^ Due to limitations in the size of the detection volume of the FCS instrument, larger pDNA polyplexes could not be accurately measured.

#### 2.6.4 Agarose Gel Retardation Assay

To evaluate the complexation efficiency of the PD block copolymer with nucleic acids, pDNA and siRNA polyplexes with different N/P ratios were prepared, as described in Section 2.4, with final pDNA or siRNA concentrations of 30 and 90 μg/mL respectively. To dissociate the nucleic acids from PD polymers, samples of 15 μL of the polyplex dispersions were mixed with 2 μL of heparin (stock 25 mg/mL in HEPES buffer 10 mM, pH 7.4) and incubated for 30 min at 37 °C, slightly modifying a previous protocol.^38^ Subsequently, 3 μL of 6× loading dye was added to each sample, and 15 μL of final polyplex dispersion was loaded into a 2.0% w/v agarose gel containing 4 μL of Midori Green. The gel was run at 100 V for 30 min in 60 mL of tris-acetate-EDTA (TAE) buffer.

The stability of the siRNA polyplexes was further evaluated with HEPES buffer 10 mM (pH 7.4) or culture medium with/without a fetal bovine serum (FBS) in a volume ratio of 1:20 (typically 10 μL of polyplexes in 200 μL of HEPES/medium). SiRNA polyplexes with a N/P of 5 were prepared, as described in Section 2.4, and incubated from 0 to 24 h at 37 °C, following the aforementioned conditions. To trigger destabilization of the polyplexes and, thus, release of the siRNA, 25 mg/mL heparin (2 μL) was used as a positive control and incubated at 37 °C for 30 min. Then, 3 μL of 6× loading dye was added to the solution, of which 15 μL (i.e., 300 ng siRNA) was loaded per well into the agarose gel. Finally, the 4.0% w/v agarose gel containing 4 μL of Midori Green was developed in 60 mL of TAE buffer and run at 60 V for 50 min. After completion, all of the gels were analyzed by a ChemiDoc Imager (Bio-Rad Laboratories Inc., Herculus, CA) using Image Lab software.

#### 2.6.5 Cryogenic Transmission Electron Microscopy

The size morphology of siRNA and pDNA polyplexes (formulated with a N/P ratio of 5) was evaluated by Cryo-TEM using a Philips Tecnai 20 (FEI/Philips Electrons Optics, Eindhoven, The Netherlands). The samples were prepared by glow-discharging the 300-mesh copper grid with a lacey carbon support (Electron Microscopy Sciences, Pennsylvania, USA) in a Cressington 208 carbon coater for 40 s. Then, 4 μL of polyplex formulation was placed onto the grid and blotted for 3–4 s in a fully automated vitrification robot (Vitrobot MARK IV, Thermo Fisher/FEI, Eindhoven, The Netherlands) at 100% humidity and room temperature. The grid was subsequently plunged into liquid ethane and stored in liquid nitrogen. Cryo-TEM images were recorded with a Gatan 626 holder (Gatan, California, USA) equipped with a 4K square-pixel Eagle CCD camera (FEI, The Netherlands). ImageJ software was used for brightness and contrast corrections of the acquired images.

### 2.7 Cell Culture

The human ovarian carcinoma cell lines (SKOV3, TOV112D, A2780, and OVCAR3) and the primary mouse embryonic fibroblasts (NIH3T3) were obtained from the American Type Culture Collection (ATCC, Rockville, MD, USA). SKOV3, TOV112D, and NIH3T3 cells were cultured in Dulbecco’s Modified Eagle’s Medium (DMEM) with high glucose (4.5 g/L glucose) supplemented with 10% fetal bovine serum (FBS) (referred to as full medium). A2780 cells were cultured in a RPMI 1640 Medium supplemented with 10% FBS. OVCAR3 cells were cultured in a RPMI 1640 Medium supplemented with 20% FBS, 1% pyruvate, and 0.01 mg/mL insulin. For the luciferase assays, the SKOV3-luc cell line, obtained from CellBioLabs (San Diego, CA, USA), was cultured in DMEM with high glucose (4.5 g/L glucose) and supplemented with 10% FBS and 1% nonessential amino acids. Cells were grown on cell culture 75 cm^2^ flasks and passaged every 3–4 days, washed with PBS, and detached with trypsin–EDTA. The cells were maintained at 37 °C in a 5% CO_2_ and humidified air atmosphere.

### 2.8 Spheroid Formation and Characterization

#### 2.8.1 Cell Viability in 2D Coculture

Prior to spheroid characterization, the cell viability of the cocultures was determined by a MTS assay and live–dead staining using Calcein AM/propidium iodide (PI) on 2D cell models. To this end, suspensions (final concentration of 7000 cells/well; 200 μL) of either SKOV3 or NIH3T3 cells were seeded in 96-well plates. Regarding cocultured cells, different ratios of cancer and fibroblast cells were used (ratio SKOV3/NIH3T3 of 5:2, 1:1, and 2:5), maintaining the same final concentration of 7000 cells/well. Then, plates were incubated at 37 °C in humidified 5% CO_2_ for 24 h. After another 24 h, 20 μL of MTS reagent was added to the wells, and cells were again incubated at 37 °C for 1 h. Read-out was performed at 490 nm (and 690 nm as a reference), using a microplate reader, SPECTROstar^Nano^ (BMG Labtech, Ortenberg, Germany). For the Calcein AM/PI staining, final concentrations of 3 and 25 μM were used, respectively. In both assays, Triton X-100 treated cells were included as a positive control.

#### 2.8.2 Spheroid Formation and Characterization

Multicellular tumor spheroids (MCTS) were prepared as homo- and heterospheroids. Homospheroids are based on only SKOV3 or NIH3T3 cells, while heterospheroids were composed of combinations thereof. For homospheroids, 400 cells/well (200 μL) of only SKOV3 or NIH3T3 cell suspensions were seeded in 96-well round-bottom Ultra-Low Attachment microplates (Corning, New York, USA). Regarding heterospheroids, different ratios of cancer and fibroblast cells were used (ratio SKOV3/NIH3T3 of 5:2, 1:1, and 2:5), maintaining the same final concentration of 400 cells/well. Then, plates were centrifuged at 350*g* for 8 min and incubated at 37 °C in humidified 5% CO_2_ for 72 h.

Following spheroid formation, MCTS cultures were analyzed with confocal laser scanning microscopy (CSLM). To this end, the ovarian cancer cells were labeled with 10 μg/mL of CellTracker Green CMFDA Dye (SKOV3, A2780, TOV112D, or OVCAR3), while NIH3T3 fibroblasts were labeled with 3 μg/mL of CellTracker Deep Red Dye (processed as pseudocolor blue) after incubation for 30 min each prior to spheroid formation. The CLSM images were recorded using a Yokogawa CV7000S imager (Yokogawa group, Tokyo, Japan) at excitation wavelengths of 488 nm for CellTracker Green CMFDA and Calcein AM and 640 nm for CellTracker Deep Red and PI (objective 20×). The images were processed using a Columbus Image Data Storage and Analysis System (PerkinElmer, The Netherlands).

To assess the versatility of the 3D platform, different homospheroid volumes of 200 μL composed of only SKOV3, A2780, TOV112D, or OVCAR3 cells (ranging from 200 to 1000 cells/well) were prepared in 96-well round-bottom Ultra-Low Attachment microplates (Corning, New York, USA). For heterospheroids, the ovarian cancer cell lines listed above were cocultured in combination with NIH3T3 cells at a 1:1 ratio and a final concentration of 400 cells/well. Plates were handled the same way as described above, and spheroids were incubated for 96 h at 37 °C before being analyzed.

### 2.9 Penetration of Polyplexes in Spheroids

**Figure 1:**
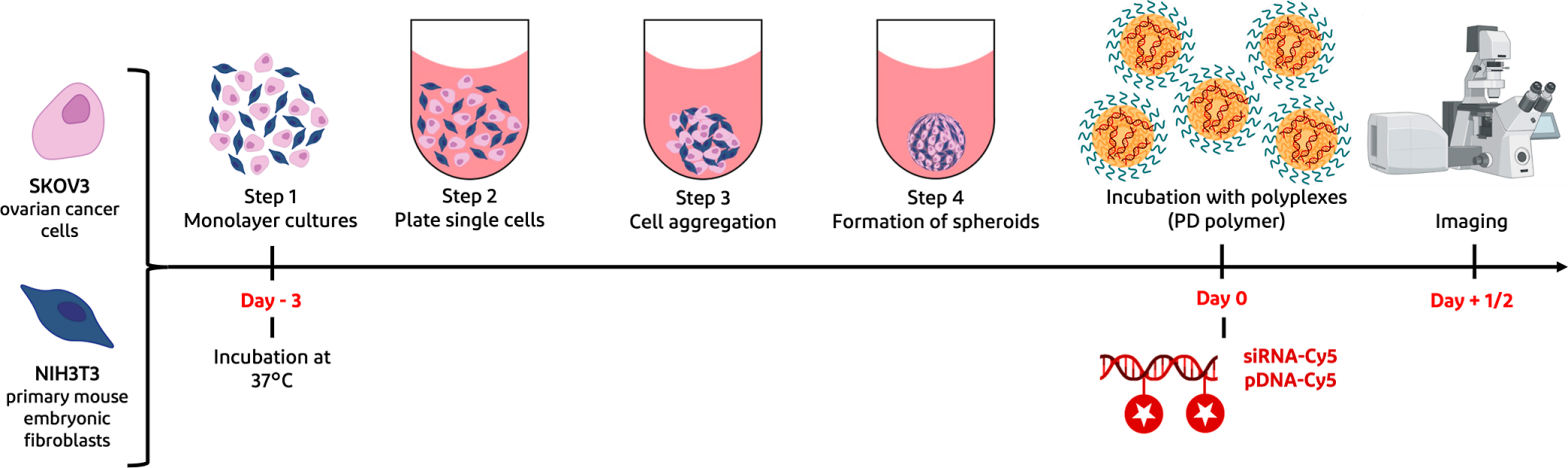
Schematic overview of experimental setup to study the penetration of polyplexes into spheroids.

Homo- and heterocultured spheroids were prepared, as described in Section 2.8.1. Both SKOV3 and NIH3T3 spheroids were stained with CellTracker Green CMFDA Dye (final concentration of 10 μg/mL). Polyplexes with N/P ratio 5, composed of pDNA-Cy5 or siRNA-Cy5, were prepared, as described in Section 2.4. For the penetration studies, 150 μL of medium in which the spheroids were cultured was removed (from a total volume of 200 μL) and subsequently 150 μL was added, composed of 130 μL of fresh medium and 20 μL of polyplex dispersions with a final concentration of 46.8 μg/mL of NA (Figure 1).

The penetration of pDNA and siRNA polyplexes into homo- and heterospheroids (ratio SKOV3/NIH3T3 of 1:1) was determined by CLSM after 24 and 48 h of incubation at 37 °C. For this, the pDNA-Cy5 and siRNA-Cy5 final concentrations in the polyplex dispersions were adjusted to 46.8 μg/mL, and the final amount of Cy5 was corrected by mixing labeled NA with nonlabeled one, resulting in the same fluorescence intensity of pDNA-Cy5 and siRNA-Cy5 per well (as described in Section 2.5). Penetration of siRNA polyplexes in homo- and heterospheroids (ratio SKOV3/NIH3T3 of 5:2, 1:1, and 2:5) was performed with a final concentration of 46.8 μg/mL.

Prior to the read-out, the spheroids were washed two times with 150 μL of PBS and subsequently, the distribution of polyplex-Cy5 fluorescence was studied by CLSM using z-stack imaging with 10 μm intervals. CLSM images were recorded using a Yokogawa CV7000S imager at excitation/emission wavelengths of 488/517 nm for CellTracker Green CMFDA and 640/666 nm for pDNA-Cy5 and siRNA-Cy5 (objective 20×). The images were processed and analyzed by using Columbus and ImageJ software. The brightness and contrast were adjusted for each channel separately using ImageJ software (HiLo LUT followed by linear adjustment of brightness and contrast).

Polyplex association, defined here as binding to and/or internalization into the cells of spheroids, was calculated as the sum of fluorescence intensities of every z-stack image recorded with 10 μm intervals up to 80 μm and corrected for the volume of the spheroid (a representative scheme can be found in Figure 6a).Figure 5

**Figure 2:**
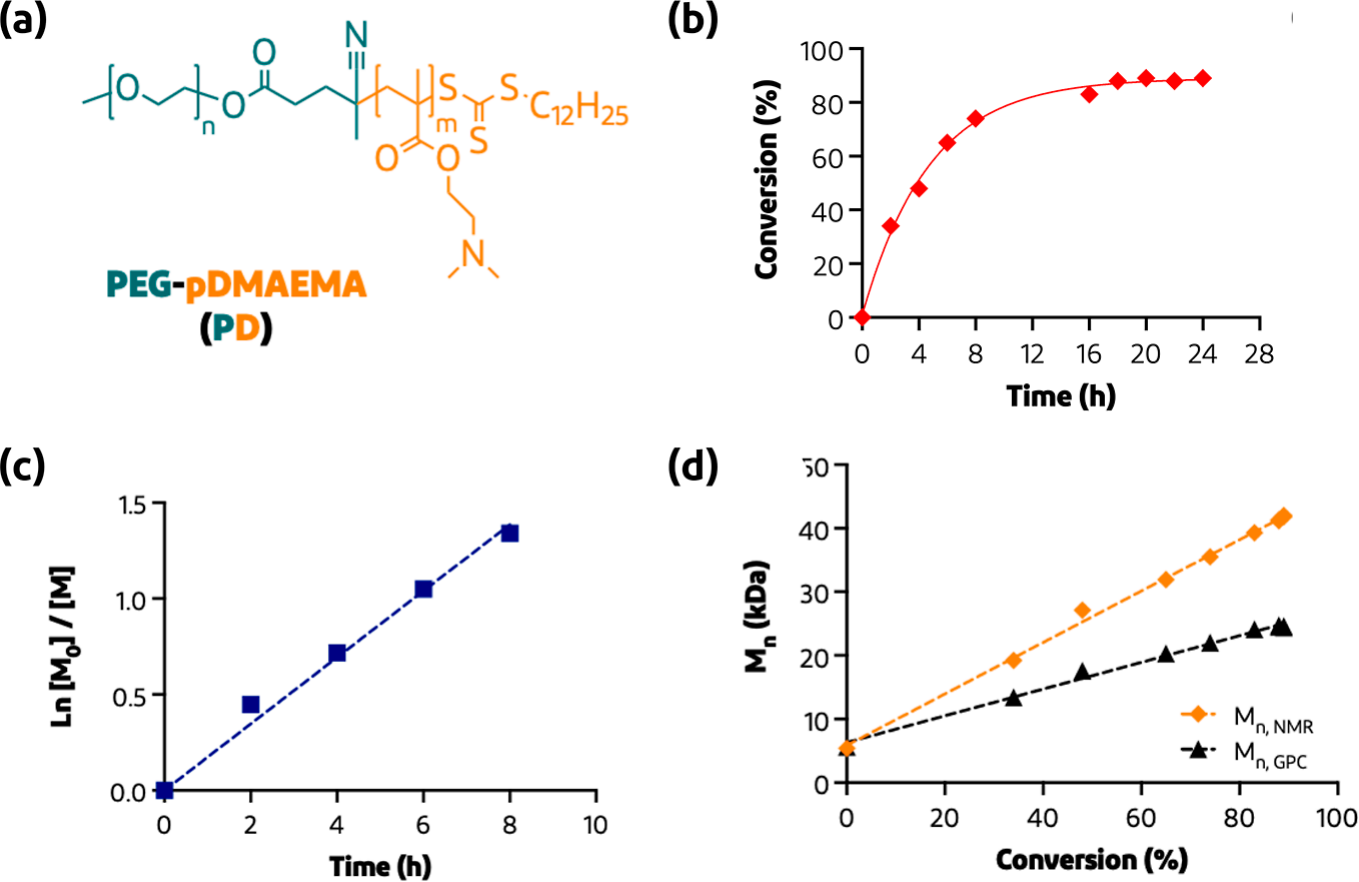
(a) Chemical structure of mPEG-pDMAEMA (PD) diblock copolymer synthesized via RAFT polymerization. (b) DMAEMA conversion measured by ^1^H NMR plotted versus polymerization time. (c) Ln[*M*_0_]/[*M*] as a function of time measured by ^1^H NMR, where [*M*_0_]= the initial concentration of DMAEMA monomer and [*M*] concentration of DMAEMA monomer in time. (d) *M*_n_ (based on GPC and ^1^H NMR analyses) versus conversion of DMAEMA.

**Figure 3:**
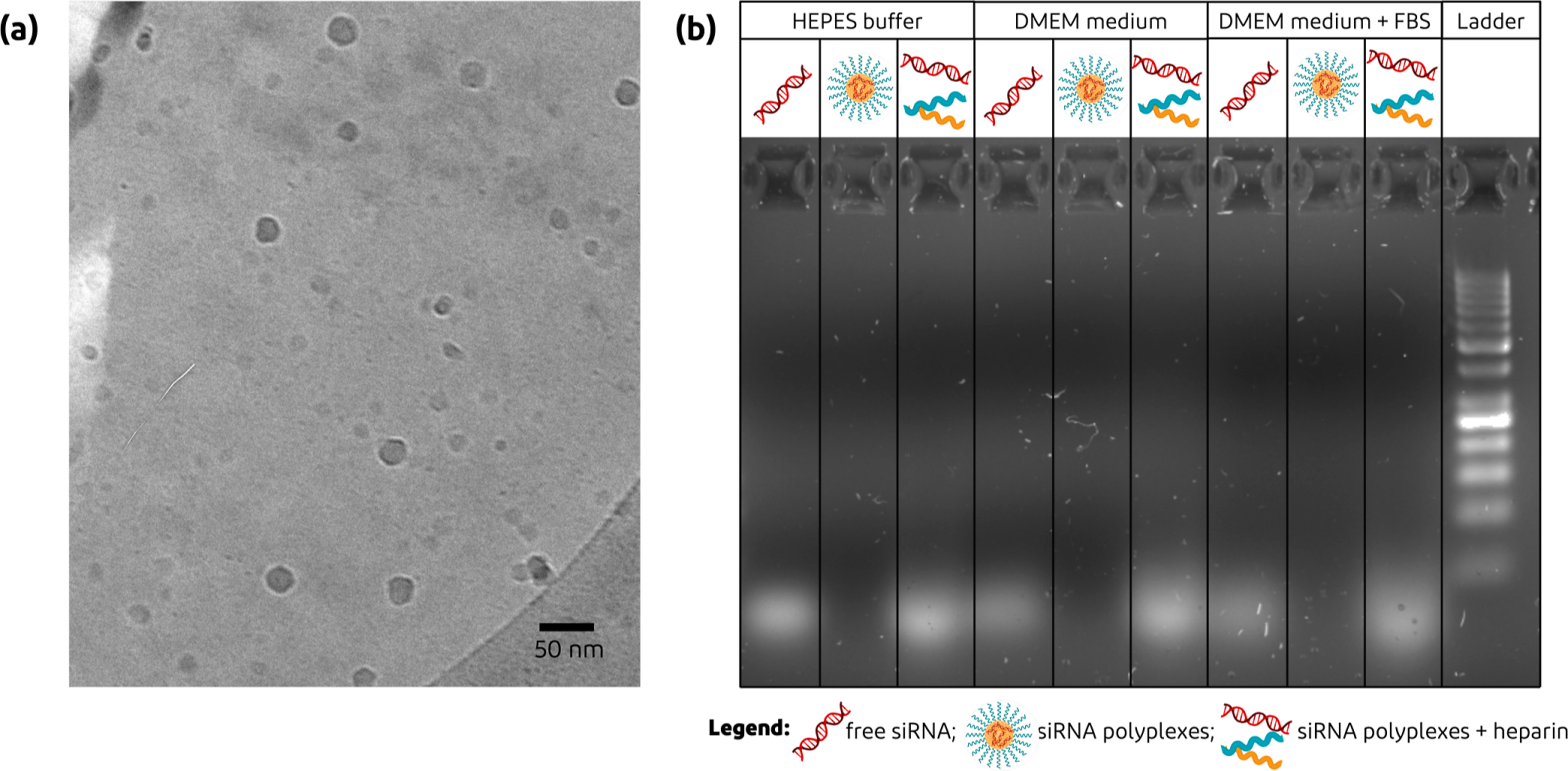
(a) Cryo-TEM image of siRNA polyplex N/P ratio 5 prepared in 10 mM HEPES buffer. (b) Agarose gel retardation assay to evaluate formation and stability of siRNA polyplex N/P ratio 5 in different media (HEPES 10 mM at pH 7.4 and DMEM medium w/o FBS).

**Figure 4:**
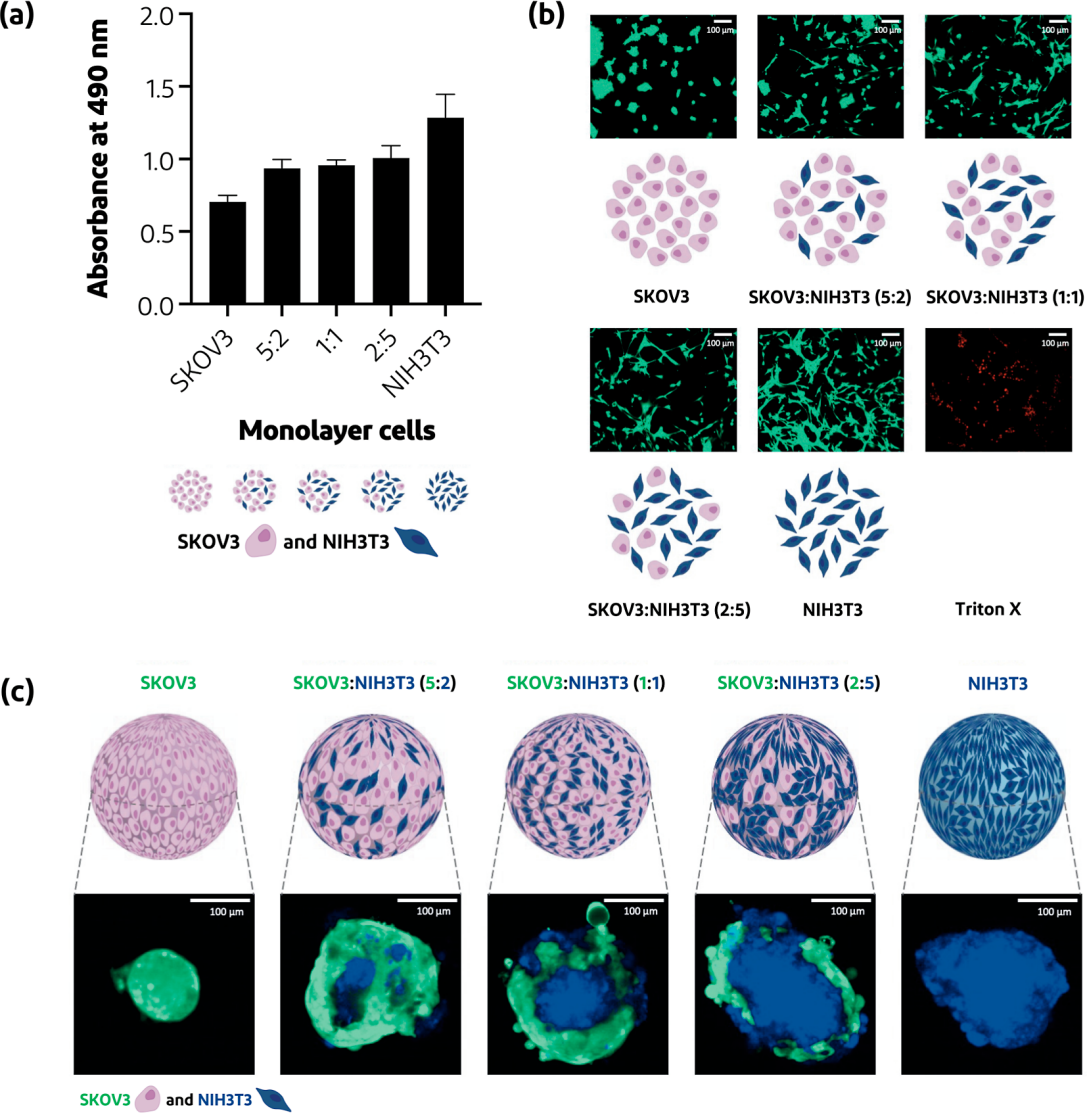
Validation of 2D cell coculture and spheroid platform. (a) Metabolic activity measured with MTS assay (expressed as absorbance) performed as 2D cell culture with SKOV3 and NIH3T3 as monocultures and cocultures (*n* = 4, mean ± SD). (b) Live–dead staining of cells cultured in 2D using Calcein AM (green, live cells) and propidium iodide (PI) (red, dead cells), respectively. (c) Schematic representation of cell ratio used for spheroid preparation (SKOV3 in pink and NIH3T3 in blue) and the actual spheroids cellular organization of homo- and heterospheroids, with SKOV3 cancer cells in green and NIH3T3 fibroblast cells in blue.

**Figure 5:**
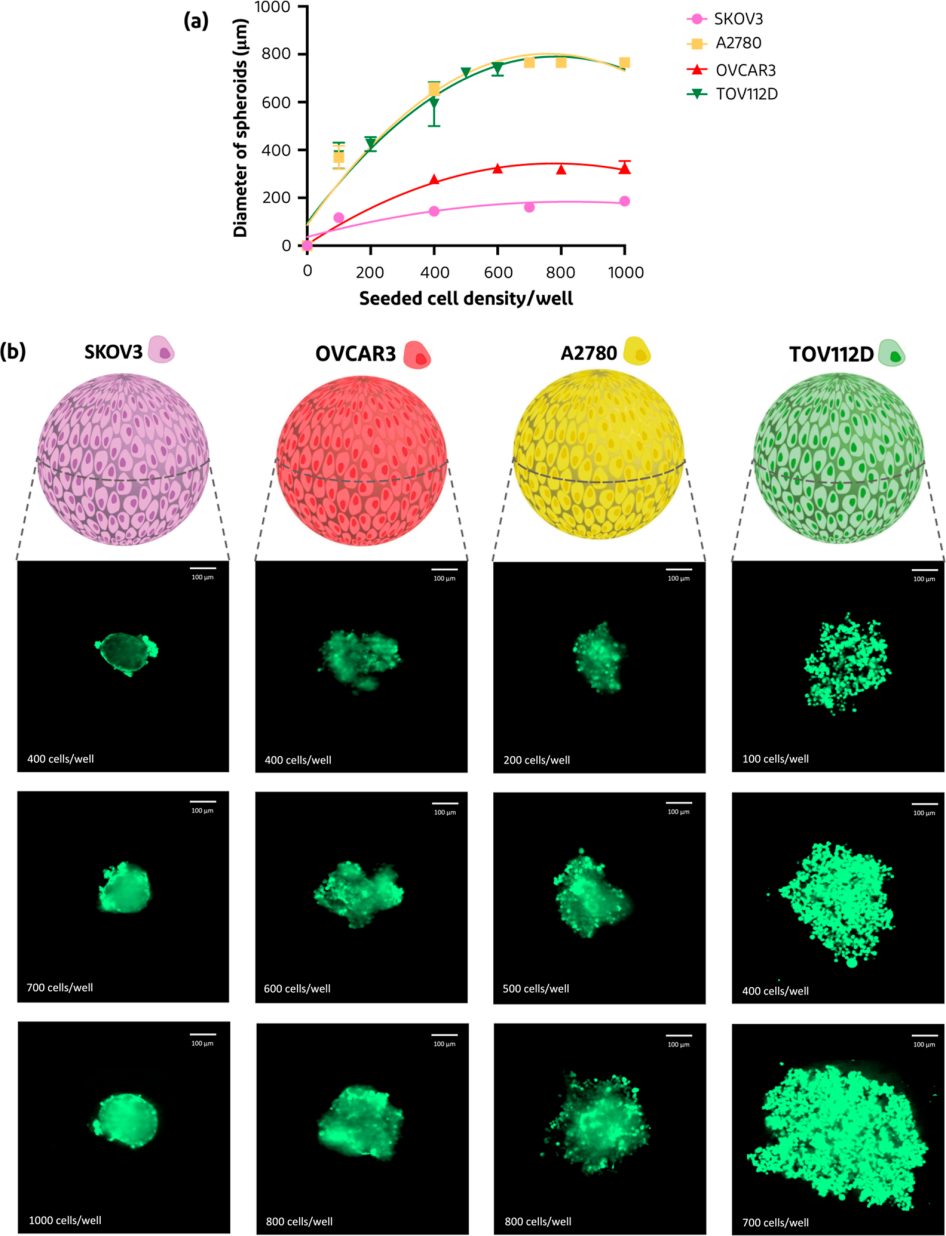
(a) Size distributions of multiple ovarian carcinoma spheroids (SKOV3, A2780, OVCAR3, and TOV112D) after seeding at different cell densities ranging from 100 to 1000 cells/well (*n* = 3, mean ± SD) after 96 h. (b) Visualization of formed spheroids after 96 h of incubation; cancer cells were stained with a green cell tracker seeded with different starting cell densities, ranging from 100 to 1000 cells/well, analyzed by CLSM (scale bars = 100 μm).

**Figure 6:**
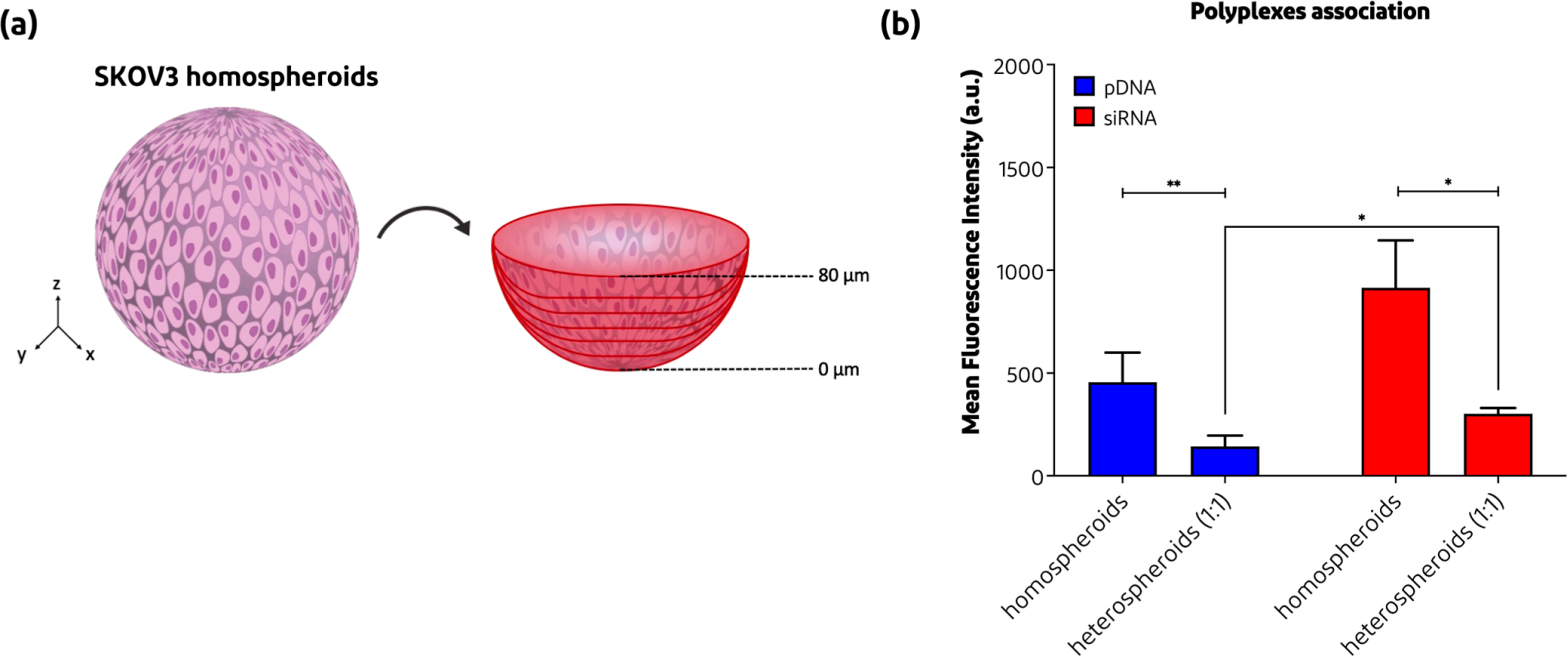
Penetration of pDNA-Cy5 and siRNA-Cy5 polyplexes into SKOV3 homospheroids and SKOV3/NIH3T3 1:1 heterospheroids. (a) Schematic overview on how the 0–80 μm region (where every z-stack image with 10 μm was summed up to 80 μm) was determined in the specific spheroids. (b) Total mean fluorescent intensity (MFI) of the pDNA-Cy5 vs siRNA-Cy5 polyplex association within the SKOV3 homospheroids and SKOV3/NIH3T3 1:1 heterospheroids after 24 h of incubation at 37 °C (*n* = 3–4), calculated as described in Section 2.9. *p < 0.05, **p < 0.01.

**Figure 7:**
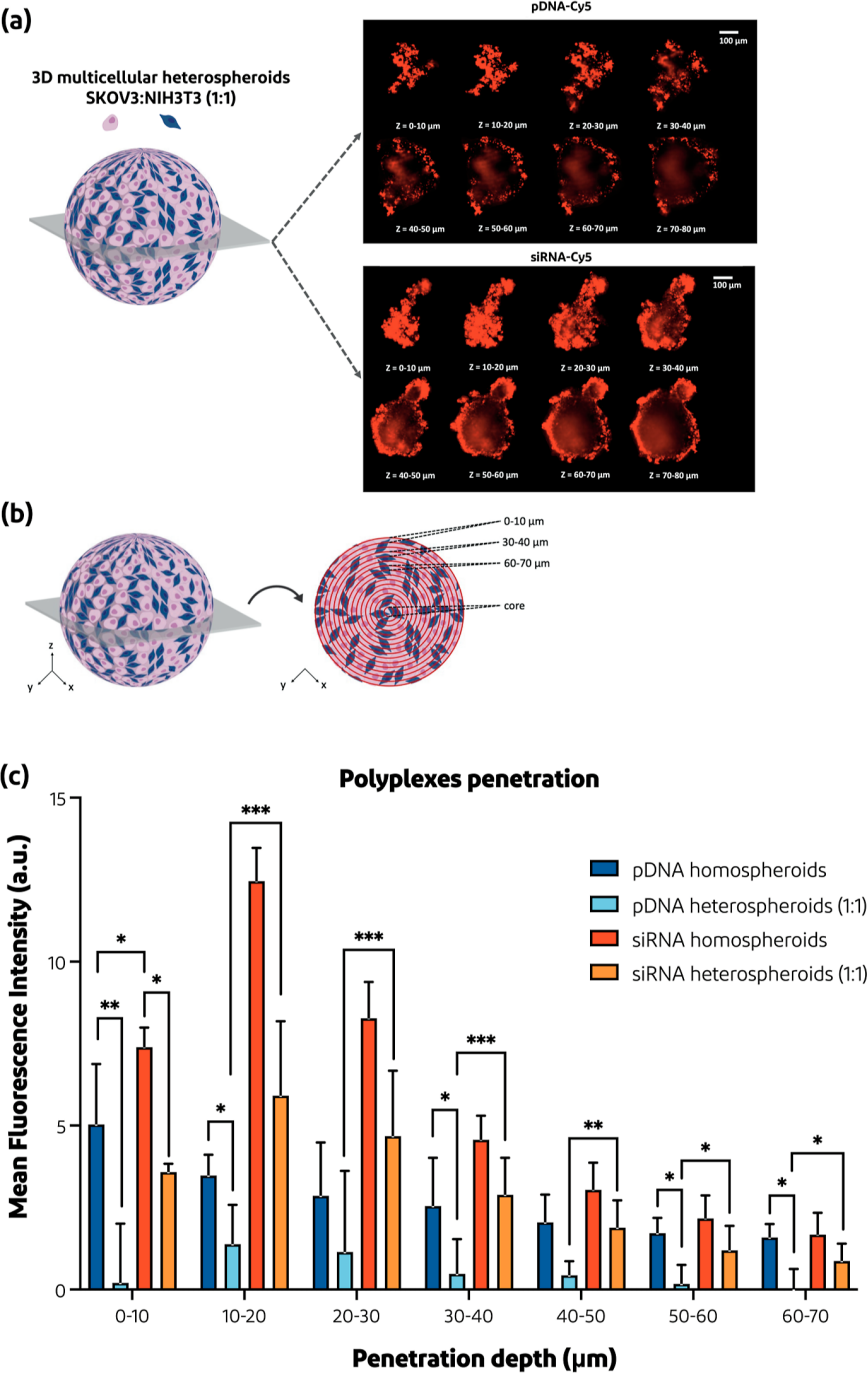
Polyplex penetration in the 8th z-stack. (a) CLSM images showing the penetration depth of pDNA-Cy5 vs siRNA-Cy5 polyplexes into SKOV3/NIH3T3 1:1 heterospheroids from the bottom up to the 8th z-stack after 24 h. (b) Schematic overview on how the 10 μm regions were determined in the specific z-stack. (c) Observed MFI per regions at the 8th z-stack (*n* = 2–5), calculated as described in Section 2.9. *p < 0.05, **p < 0.01, and ***p < 0.001. Scale bar = 100 μm.

For penetration depth analysis along the x*y*-axis of a specific z-stack, regions of interest of 10 μm were created with Analyze Particles and Enlarge tools from ImageJ (representative scheme can be found in Figure 7b). Next, mean fluorescence intensity (MFI) was measured per region, and subsequently normalized and corrected for the area.

### 2.10 Cell Internalization of siRNA Polyplexes in 2D Mono- and Cocultures

To investigate the mechanisms of siRNA polyplex penetration, cellular uptake studies were performed on the monoculture SKOV3-luc and NIH3T3 cells and on the cocultured cells at a ratio SKOV3-luc/NIH3T3 of 3:2. In order to distinguish the two cell lines in the coculture, either SKOV3-luc or NIH3T3 cells were prestained with the CellTracker Green CMFDA following the method, as described in Section 2.8.2. After that, the cells were seeded into a 96-well μClear black plate with a final concentration of 7000 cells/well and incubated for 24 h at 37 °C in full medium. Then, the cells were incubated with siRNA-Cy5 polyplexes (N/P ratio 5 with a final siRNA concentration of 375 nM per well) and incubated for 4 and 24 h at 37 °C. Before imaging, the cells were washed twice with PBS. CLSM images were recorded using a Leica TCS SP8 X (Leica, Amsterdam, The Netherlands) microscope at excitation wavelengths of 405, 488, and 561 nm for Hoechst 33342, CellTracker Green CMFDA, and siRNA-Cy5, respectively (objective lens 10×).

### 2.11 In Vitro Cytotoxicity Studies and Luciferase Assay in 2D Cell Layout

To assess the cytotoxicity of the siRNA polyplexes, SKOV3-luc cells were seeded in a 96-well plate (concentration of 7000 cells/well) in a full medium and cultured for 24 h at 37 °C in a 5% CO_2_ and humidified air atmosphere. Subsequently, the cells were incubated with 200 μL of siRNA polyplexes with a N/P ratio of 5 and at siRNA final concentrations (ranging from 100 to 500 nM per well) for 24 h at 37 °C in the full medium. Then, the medium was replaced with a fresh full medium, and the cells were incubated at 37 °C for another 24 h. After that, 20 μL of MTS assay reagent was added for each well. After 1 h of incubation at 37 °C, the read-out was performed at 490 nm (and 690 nm as a reference), using a microplate reader SPECTROstar^Nano^ (BMG Labtech, Ortenberg, Germany) and the cell viability was calculated following eq 2
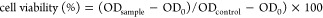
2where OD_sample_, OD_control_, and OD_0_ are the optical density (OD) values of the medium of transfected cells, the medium of untreated cells, and only medium (as a background), respectively. The transfection experiments were conducted using a Lipofectamine 3000 as a commercially available transfection agent (following manufacturer’s protocol) and noncoding luciferase siRNA as the negative control.

Silencing efficiency of the siRNA polyplexes was assessed with a luciferase assay and performed in parallel on a separate fully white plate. The transfection protocol was similar to that described above, but instead of adding MTS reagent, lysis buffer of the luciferase assay was added, and luminescence was measured as read-out by a GloMax Discover System (Promega, Leiden, The Netherlands).

### 2.12 Statistical Analysis

The *p* values were determined by a Student’s test with two-tailed distribution performed with the software GraphPad Prism 9 (GraphPad Software Inc., La Jolla, California). *p* values < 0.05 are statistically significant.

## 3 Results and Discussion

### 3.1 Polymer Synthesis and Characterization

In this study, a mPEG-CTA (P) polymer, with a methoxy group on one terminal end and bearing a thiocarbonylthio functionality (CTA) on the other end, was used for reversible addition–fragmentation chain-transfer (RAFT) polymerization of DMAEMA (D), according to a slightly modified reported protocol (Figure 2a).^34^ The polymerization kinetics of DMAEMA were investigated for 24 h and shown in Figure 2b. For the first 8 h, the conversion of DMAEMA (up to 80%) showed a linear relationship of ln[*M*_0_]/[*M*] over time (Figure 2c) demonstrating first-order kinetics.^39,40^Figure 2d shows the number-average-molecular-weight (*M*_n_) as a function of time measured by GPC and ^1^H NMR. Both analytical methods showed that the *M*_n_ of mPEG-pDMAEMA (PD) increased linearly with the conversion. To be mentioned, the *M*_n_’s obtained via GPC displayed lower values compared to the ones measured by NMR, which can be related to the use of PEGs as GPC standards, showing different hydrodynamic volumes compared to the PD diblock copolymer when dissolved in the same solvent.^34^ The obtained *M*_n_ of the mPEG-pDMAEMA diblock copolymer was 35.2 kDa with a *D̵* of 1.44 (^1^H NMR spectrum in Figure S1, Supporting Information), which is close to the aimed value selected based on the balance between toxicity and transfection properties.^34,41,42^ Moreover, the mPEG-pDMAEMA polymer can be removed from the body by excretion via renal filtration (considering that its *M*_n_ is lower than a renal cutoff of ∼50 kDa) and/or by hepatic clearance.^43^

### 3.2 Polyplex Characterization

The synthesized mPEG-pDMAEMA (PD) diblock copolymer was used to prepare polyplexes containing either pDNA or siRNA as cargo, with varying N/P molar ratios from 1 to 10. DLS analysis showed that polyplexes prepared with pDNA had diameters ranging from 139 to 165 nm (Table 1). On the other hand, siRNA polyplexes did show a significant decrease in particle diameter from 185 ± 6 nm at N/P 1 to 25 ± 2 nm at N/P 5. This decrease in size can be explained by considering that at a high N/P ratio of 5, the siRNA molecules are more diluted compared to the N/P 1 ratio. It is highly likely that they form polyplexes with only a few siRNA molecules, as already hypothesized previously.^42,44^ Overall, the smaller size of the siRNA polyplexes can be explained by the smaller molecular weight of siRNA compared to pDNA (13.3 and 3416.4 kDa, respectively). Findings regarding these sizes of both pDNA and siRNA polyplexes are in line with previous studies.^41,42^ Further decrease in particle sizes with an increase in the ratio to N/P 10 for siRNA polyplexes was not observed. Both pDNA and siRNA polyplexes showed a narrow size distribution falling in a range of 0.1–0.3, regardless of the type of cargo. Polyplexes with N/P ratio 1 showed negative ζ-potential values, whereas the ones prepared at higher N/P ratios showed positive values for both pDNA and siRNA polyplexes (Table 1). This can be explained by the large excess of positively charged nitrogen atoms of cationic PD diblock copolymer compared to the negative charges of the nucleic acids.^45−47^ Indeed, polyplexes based on cationic polymers and pDNA/siRNA and prepared at a high N/P ratio have a zeta potential of +20–30 mV.^16,48,49^ The siRNA and pDNA polyplexes prepared at N/P ratios of 5 and 10 showed a zeta potential of +7–8 mV, which can be ascribed to the shielding of the surface charge of the polyplexes by PEG as also found in other studies.^18,50,51^

**Table 1:** Characteristics of pDNA and siRNA Polyplexes Prepared with Different N/P Ratios Ranging from 1 to 10 in HEPES Buffer (10 mM, pH 7.4)^a^

formulation	N/P ratio	diameter (nm)	PDI	ζ-potential (mV)
pDNA polyplexes	1	169 ± 7	0.13 ± 0.05	–13.9 ± 0.5
	5	162 ± 11	0.16 ± 0.02	6.8 ± 0.5
	10	139 ± 8	0.15 ± 0.01	8.4 ± 0.3
siRNA polyplexes	1	185 ± 6	0.03 ± 0.02	–7.5 ± 0.9
	5	25 ± 2	0.35 ± 0.05	7.8 ± 0.3
	10	27 ± 3	0.31 ± 0.04	8.1 ± 0.5

a Hydrodynamic diameter and polydispersity index (PDI) were determined by dynamic light scattering (DLS) at 37 °C, whereas the ζ-potential of the particles was measured by laser doppler electrophoresis (LDE) at 37 °C (*n* = 3, mean ± SD) in the same medium.

The size and the resulting loading capacity of siRNA polyplexes were analyzed by FCS, using fluorescently labeled siRNA-Cy5. Plots of the FCS measurements are shown in Figure S2, Supporting Information. Table 2 shows that a decrease in the number of NA molecules per polyplex from 63 to 3 was observed with an increase in the N/P ratio from 1 to 10. The decrease in size with increasing N/P of polyplexes as determined by FCS is in agreement with the trend obtained with DLS. Differences in size numbers observed for the N/P 1 and 10 polyplexes recorded with both FCS and DLS can be attributed to the use of different techniques. In case of the presence of a few larger particles, which scatter the light much more than small particles, DLS measurements may overestimate the average size of the particles.^52^ It should be noted that also FCS may be biased toward few larger particles that contain more fluorophores. As seen from the FCS time traces in Figure S2 (Supporting Information), the height and polydispersity of fluorescence peaks decreases with increasing N/P ratio, confirming that more uniform and smaller polyplexes were formed. Cryo-TEM measurements were performed to study the size and morphology of the polyplexes (while for pDNA polyplexes an additional a SEM image can be found in Figure S3, Supporting Information). As shown in Figure 3a, particles with an N/P ratio of 5 with a spherical shape were observed with diameters falling in the range of 20–40 nm, which is in line with DLS and FCS measurements. The stability of polyplexes was assessed using an agarose gel retardation assay, showing that both the pDNA and siRNA polyplexes prepared at N/P ratio 5 quantitatively complexed the NAs and no free NAs were observed (Figure S4, Supporting Information). Heparin was used to destabilize the polyplexes and trigger the release of the NAs. Based on this finding, in the following experiments, the siRNA polyplexes were exclusively formulated with an N/P ratio of 5, which represents the minimal amount of nitrogen groups required to confer stable particles. To notice, another study demonstrated that siRNA polyplexes, which were based on the same PD diblock copolymer and formulated at an N/P ratio of 3, displayed unstable and large particle sizes with a high PDI, despite their ability to retain the siRNA according to the agarose gel assay.^42^ For consistency, pDNA polyplexes with an N/P ratio of 5 have been investigated to maintain the same N/P ratio as that of the siRNA polyplexes. Furthermore, to examine the stability of polyplexes, siRNA polyplexes prepared at the N/P ratio 5 were incubated under different conditions (HEPES 10 mM pH 7.4 and in a DMEM medium with and without FBS). Under these incubation conditions, no free siRNA was detected indicating that the cationic PD diblock copolymer was able to form stable complexes with the siRNA even in media with FBS (Figure 3b).

**Table 2:** Polyplex Diameters and siRNA Loading Capacity Analyzed with FCS^a^

formulation	N/P ratio	diameter (nm)	average number of siRNA molecules per complex
free siRNA	n.a	2 ± 0	n.a
siRNA polyplexes	1	62 ± 2	63 ± 27
	5	36 ± 3	5 ± 1
	10	12 ± 1	3 ± 1

a Free siRNA-Cy5 was included as the control (n = 3, mean ± SD). The complete set of calculations is described in Table S2, Supporting Information n.a. = not applicable.

### 3.3 Formation and Characterization of Spheroids

Looking at the ability of the SKOV3 ovarian cancer cells to form moderately differentiated adenocarcinomas in vivo, they have been selected as a cell line model for the formation of the spheroids.^53,54^ To develop the spheroid platform, we first assessed whether ovarian carcinoma cells (SKOV3) could be cocultured with primary mouse embryonic fibroblasts (NIH3T3). This way, the cocultured cells can mimic the in vivo ovarian tumor nodules, including the ECM. Indeed, as previously shown by Priwitaningrum et al., by increasing the NIH3T3 cell concentration the percentage of collagen-1 increases proportionally.^31^Figure 4 shows that when performing an MTS assay on 2D monocultures the absorbance at 490 nm for the NIH3T3 cells was higher than that for the SKOV3 cells. The absorbances of the cocultures were in between the values for the monocultures and increased with NIH3T3 content, indicating that SKOV3 and NIH3T3 cells can coexist. To confirm this finding, live–dead staining was assessed by CLSM. Regardless of all mono- and cocultures with different ratios, Calcein AM (green) was solely recorded, confirming the presence of live cells (Figure 4). Moreover, no red color related to the intracellular presence of propidium iodide was observed in the mono- and cocultures, showing that SKOV3 and NIH3T3 can indeed coexist without affecting each other’s cell viability, as shown in Figure 4. Both cell lines preserved their characteristic cellular morphology, which was rounded for SKOV3 and elongated for NIH3T3 (Figure 4).

Having shown that SKOV3 can be cocultured with NIH3T3 in 2D culture, spheroids were formed, and their cellular organization was investigated. To mention, one of the main advantages of this new platform is the absence of Matrigel which, given its high variability of each batch, can lead to a lack of reproducibility and issues to harvest spheroids, limiting accurate effectiveness and controlled size of the spheroids.^55^ Moreover, this in vitro method offers multiple advantages compared to other existing matrix-independent and matrix-dependent techniques such as easily scalability, reproducibility, fast-screening, and low cost.^56^ Spheroids with different ratios were examined using CLSM, where SKOV3 cells and NIH3T3 cells are colored green and blue, respectively. The heterospheroids showed that cells of the same type prefer to self-assemble with each other as observed by the clustered green and blue areas (Figure 4), which is in line with previous studies with other cell types.^57,58^ Simultaneously, it was observed that spheroids of cocultured NIH3T3 and SKOV3 cells were bigger in size than monocultured SKOV3 spheroids only (diameter of ∼259 vs ∼132 μm), showing a trend of metabolic activity similar to that of the MTS assay (Figure 4). Moreover, to prove that the spheroids developed are fully packed with cells (even in the core) without a dip in their shape, histological, and confocal characterization of SKOV3 homospheroids was performed (Figure S5, Supporting Information).

To further establish the versatility of such 3D in vitro spheroid models for ovarian cancer, different ovarian carcinoma cell lines were investigated. SKOV3, A2780, TOV112D, and OVCAR3 cells are some representative cell lines of the many ovarian cancer cell lines that are commonly used in in vitro studies, of which the first two are cited the most.^59,60^ To check if the 3D in vitro spheroid platform is versatile and can be exploited with all these cell lines, we also analyzed different starting cell densities. Figure 5a shows that homospheroids of SKOV3 resulted in the smallest spheroids (100–200 μm), while A2780- and TOV112D-based spheroids displayed larger sizes of 300–800 μm. When looking at the acquired CLSM images, homospheroids of all ovarian carcinoma cell lines grew larger when seeding at higher cell densities (Figure 5b), which is in line with previous studies.^61^ When all four cell lines were compared with each other, SKOV3 and OVCAR3 seem to form the smallest homospheroids, which could be explained by a low metabolic activity. On top of that, it seems that A2780 and TOV112D spheroids are less dense compared to the OVCAR3 and SKOV3 spheroids, as more gaps are visible between the A2780 and TOV112D cells (Figure S6a, Supporting Information). This could be due to the growth time of the spheroids, where certain cell lines would need more time to grow into tight spheroids and therefore reach a higher solidity. So, extending their growth time could possibly result in a tighter spheroid. Similar to for SKOV3/NIH3T3 spheroids, during culturing of heterospheroids composed of other ovarian cancer with NIH3T3, cells of the same type prefer to self-assemble with each other as the green and red colors are clustered (Figure S6b, Supporting Information). Finally, when looking at the sizes of the 1:1 heterospheroids of the different ovarian cancer cell lines, it can be observed that SKOV3-based heterospheroids again correspond to the smaller spheroids, compared to the A2780 and TOV112D heterospheroids (Figure S6c, Supporting Information). Importantly, in vivo ascitic tumors generally range in a size of 100–700 μm,^62,63^ which is close to our spheroids, making them suitable model systems to study penetration of nanoparticles into ovarian nodules. The combination of the tailorable size of the spheroids with the possibility of coculturing them in the presence of other cells (i.e., fibroblasts) offer an important tool for the screening of different treatments. Not least, the use of this platform avoids the use of fixation/sectioning and the clearing process of spheroids, which are both time-consuming techniques. For this, the purposed fast-screening tool can help to compare different polyplex penetrations and find the most suitable formulation for preclinical applications (reducing the number of animals for in vivo tests).

### 3.4 Penetration of Polyplexes in Spheroids

The association and penetration of both pDNA and siRNA polyplexes into the spheroids were investigated with CLSM. For this study, only homospheroids SKOV3 of ovarian cancer cells and heterospheroids of these cells cocultured with NIH3T3 cells (at ratios 5:2, 1:1, and 2:5) were used. For the live imaging of the spheroids, the optical cross sections for examination were established by first determining the bottom of the spheroid and then visualizing z-stacks higher, whereby one z-stack corresponds to 10 μm (Figure 6a). By measuring the total mean fluorescent intensity (MFI) of the acquired images, it was observed that siRNA polyplexes (particle size of ∼25 nm at N/P 5) showed an association within the SKOV3 homospheroids of 2 times higher than pDNA polyplexes (particle size of ∼162 nm at N/P 5) after 24 h of incubation (Figure 6b). Comparing the polyplex fluorescence intensities of the SKOV3 homospheroid association with the SKOV3/NIH3T3 1:1 heterospheroids, it is observed that the presence of the fibroblasts negatively influenced the polyplex association. More specifically, the MFI decreased from 456 ± 144 to 143 ± 53 for the pDNA polyplexes while for siRNA polyplexes the decrease of the MFI was from 915 ± 230 to 302 ± 28. Nevertheless, also for the heterospheroids, the siRNA polyplexes showed higher around two times higher association than the pDNA polyplexes.

The penetration depth of the polyplexes into the 1:1 SKOV3 homospheroids and 1:1 SKOV3/NIH3T3 heterospheroids was quantified at the height of the eighth z-stack. Figure 7a shows that the siRNA polyplexes penetrated deeper and even reached the center of the eighth z-stack of the spheroids as compared to the pDNA polyplexes, which were mainly located in the rim. This difference in the penetration area is in line with previous studies in which it was shown that the penetration of nanoparticles based on lipids, polymers, and silica in spheroids and even tumors was size-dependent.^22,23,64−67^ Furthermore, we were able to establish the regional penetration pattern for both siRNA and pDNA polyplexes, as schematically represented in Figure 7b. As shown in Figure 7c, the siRNA polyplexes have substantially better penetration into both homo- and heterospheroids than the pDNA polyplexes, even when the spheroids were cocultured with fibroblasts, indicating the ability of siRNA polyplexes to overcome the ECM produced by the fibroblasts, likely due to their small particle size.

The effect of the incubation time of both pDNA and siRNA polyplexes on their penetration into SKOV3 homospheroids was investigated. Remarkably, incubating the homospheroids with polyplexes for 48 h instead of 24 h did not enhance the total MFI of the spheroids (Figure S7, Supporting Information).

**Figure 8:**
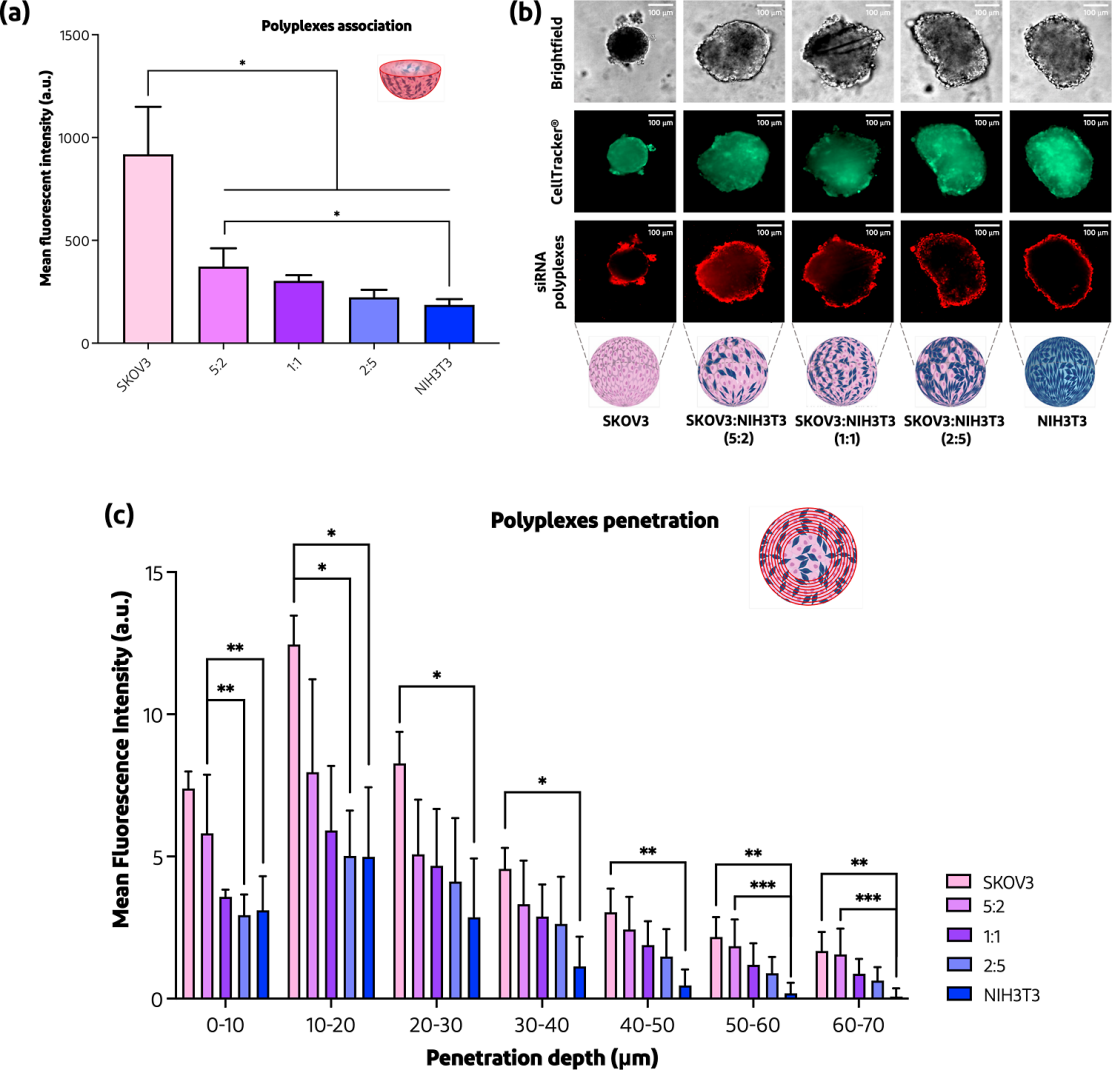
Penetration of siRNA polyplexes into SKOV3/NIH3T3 homo- and heterospheroids. (a) Total mean fluorescent intensity (MFI) of the siRNA-Cy5 polyplexes into homo- and heterospheroids after 24 h (*n* = 6–8), calculated as described in Section 2.9. (b) CLSM images showing the penetration at the 8th z-stack of siRNA-Cy5 polyplexes into homo- and heterospheroids (5:2, 1:1, and 2:5) after 24 h. (c) MFI per regions regarding the specific z-stack of spheroids from 0 to 70 μm (*n* = 5–8), calculated as described in Section 2.9. *p < 0.05, **p < 0.01, and ***p < 0.001. Scale bar = 100 μm.

Further, we evaluated the influence of NIH3T3 on penetration rate of siRNA polyplexes, by using spheroids based on both homo- and heterospheroids at different ratios, 5:2, 1:1, and 2:5 (SKOV3/NIH3T3). Figure 8a shows how the total MFI found in the SKOV3 homospheroids incubated with siRNA polyplexes was significantly higher than that observed for the heterospheroids. Moreover, with increasing the fibroblast content, the MFI decreased, as visualized also by CLSM in Figure 8b. As can be seen in Figure 8c, the penetration depth of siRNA polyplexes was also dependent on the fibroblast content of the spheroids because these cells produced ECM to a much higher extent than tumor cells only. Importantly, even though the penetration of siRNA polyplexes is strongly affected by ECM produced by the fibroblasts, the nanoparticles can still reach the first rings of the ovarian nodulus, with a penetration depth of ∼60–70 μm. Overall, the polyplex penetration ability is similar among the heterospheroids tested, regardless of the concentration of fibroblasts seeded. Translating our findings into an in vivo scenario, the siRNA polyplexes proved to be a potential candidate for the penetration and eradication of small tumor nodules. For eradication of bigger nodules, a synergistic co-medication and/or ECM weakening adjuvant agent may be required.

### 3.5 Cell Internalization of siRNA Polyplexes by Cells in 2D Coculture

**Figure 9:**
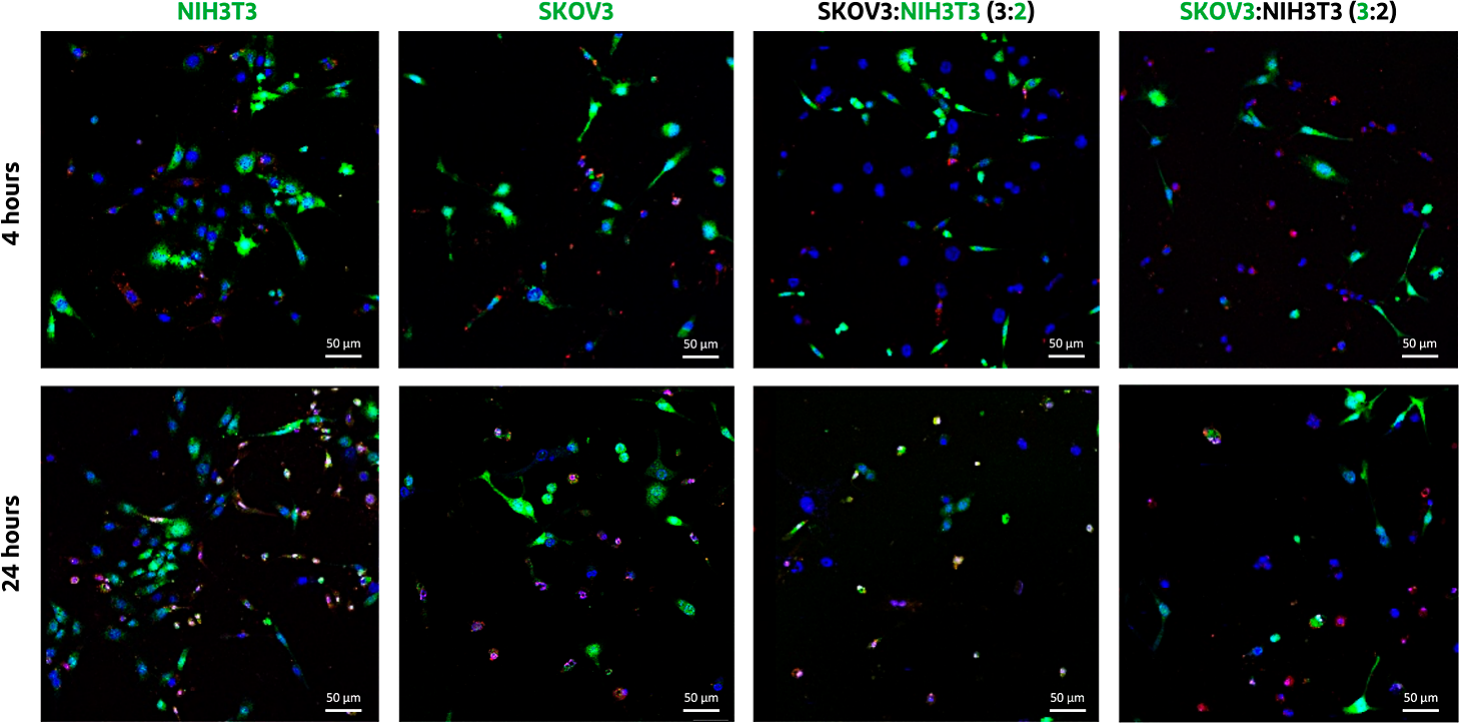
CLSM images of monoculture SKOV3-luc and NIH3T3 cells, and the coculture SKOV3-luc/NIH3T3 (ratio of 3:2) after 4 and 24 h of incubation with siRNA-Cy5 polyplexes at 37 °C in full medium. Cell Tracker corresponds to the green color (CellTracker Green CMFDA Dye), cell nuclei to blue (Hoechst 33342) and siRNA to red (siRNA-Cy5). For improved readability, in the SKOV3-luc/NIH3T3 ratio 3:2 condition, both cell types were alternatively stained with the CellTracker Green CMFDA Dye. First, NIH3T3 cells were stained (middle-right side), followed by staining of only the SKOV3-luc cells (depicted on the right side).

To better understand how the siRNA-Cy5 polyplexes are able to penetrate within the spheroids even in the presence of fibroblasts, we investigated their cellular uptake in monoculture SKOV3-luc and NIH3T3 cells, as well as in the coculture SKOV3-luc/NIH3T3 cells at a 3:2 ratio using CLSM. As can be noticed in Figure 9, regardless of the incubation time of the particles, after 4 or 24 h of incubation, the siRNA- Cy5 polyplexes of N/P 5 ratios were present intracellularly in all tested conditions. This indicates the versatility of pDMAEMA as a nonviral gene carrier capable of improving the cellular uptake of siRNA not only within SKOV3-luc cells but also within fibroblasts, as confirmed by other studies.^13,68^ Looking at the mechanism of internalization, in general siRNA polyplexes of ∼100 nm featuring cationic properties are taken up via clathrin-mediated endocytosis, whereas smaller siRNA polyplexes (<50 nm) are usually internalized through caveolin-mediated endocytosis.^69,70^ Therefore, it is likely that the siRNA polyplexes will be internalized via the caveolin-mediated endocytosis pathway. However, not all cells are equipped with these proteins, so further investigation should be carried out in order to provide a detailed explanation of the internalization pathway of siRNA polyplexes. Overall, both punctuate fluorescence (arrow heads) as diffuse cytosolic signal (arrows) was observed in both SKOV-luc and NIH3T3 cells (Figure S8, Supporting Information). Most likely, the siRNA-Cy5 polyplexes are being internalized inside the cells by endocytosis, after which part of the complexes are present inside the endosomes, following the pathway of trafficking and entrapment inside these vesicles as previously described,^71,72^ while a fraction of siRNA is able to escape the endosomes into the cytosol. To be mentioned, free siRNA was not internalized because siRNA is a rather high-molecular-weight, hydrophilic, and negatively charged molecule unable to spontaneously pass cellular membranes (Figure S9, Supporting Information).^10,73,74^ Taken together, the envisioned mechanism of polyplex penetration inside the spheroids seems to be related not only to the passive diffusion of the particles, which can be limited in some cases due to the compact nature of spheroids and presence of ECM (Figures 7 and 8) but also to the cellular uptake and potential intercellular transport of the polyplexes within the spheroids, as Figure 9 highlights.

### 3.6 Cell Viability and Luciferase Silencing in 2D Cell Culture upon Incubation with siRNA Polyplexes

**Figure 10:**
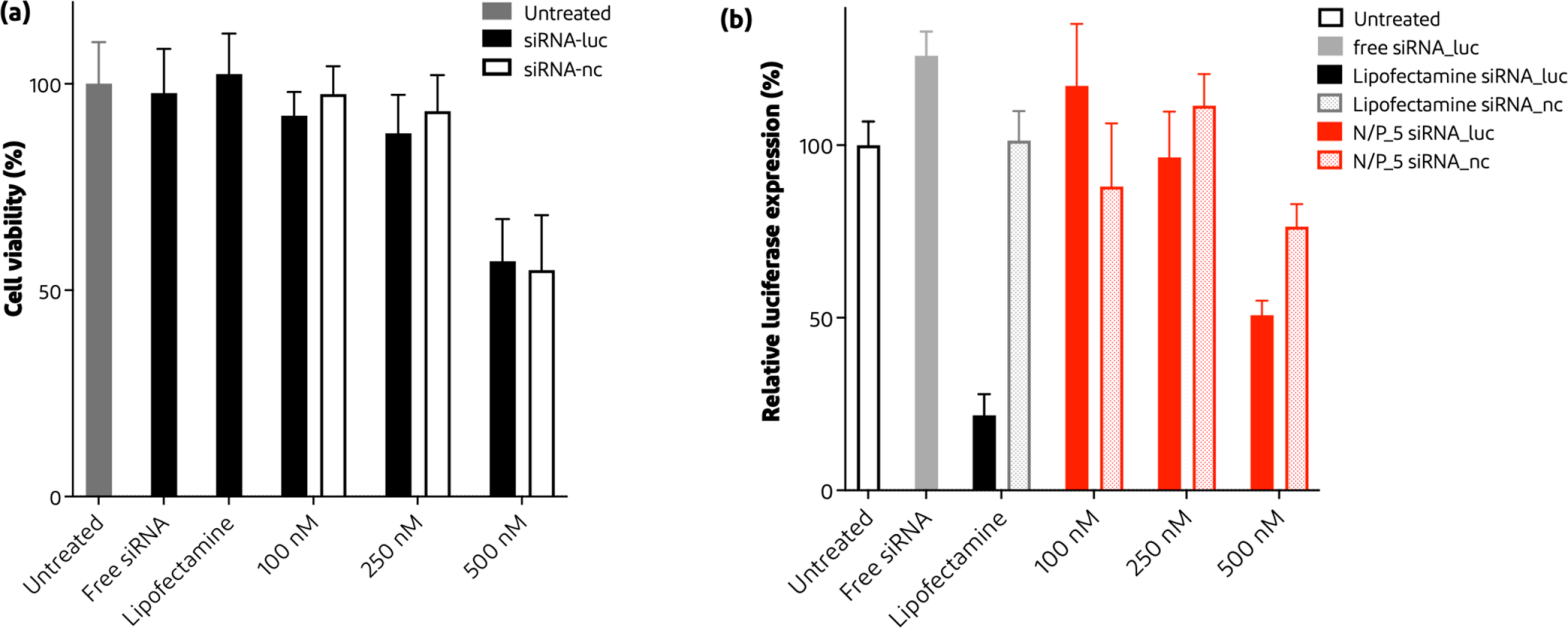
Cytotoxicity and luciferase silencing efficiency of siRNA polyplexes on SKOV3-luc cells. (a) Metabolic activity of SKOV3-luc cells determined using MTS assay upon incubation for 24 h at 37 °C with siRNA polyplexes at N/P ratio 5 and concentrations ranging from 100 to 500 nM, while free siRNA and Lipofectamine with 100 nM final concentration (*n* = 10–15). (b) Luciferase expression was measured after 24 h incubation with siRNA polyplexes prepared at N/P 5. Similar concentrations were used as in the cytotoxicity study, whereby noncoding siRNA (siRNA-nc) was included as a negative control (*n* = 5–7).

Following the cell uptake studies, the cytotoxicity and silencing efficiency of the siRNA polyplexes (encoding for luciferase and noncoding) were examined using SKOV3-luc cells. Increasing the concentration of polyplexes resulted in a lower cell viability (Figure 10a), indicating that polyplexes are cytotoxic likely due to the cationic nature of the PD diblock copolymer, as also observed for other cationic polymers.^75^ However, a preliminary in vivo study of siRNA polyplexes based on the PD diblock copolymer indicated their safety for preclinical applications.^76^Figure 4Figure 10b shows the silencing efficacy of different siRNA polyplex formulations. Noncoding siRNA polyplexes (siRNA-nc, dotted bars) did not silence the luciferase expression of SKOV3-luc cells (Figure 10b). To study sequence specific downregulation, luciferase expression by cells was measured upon incubation with a medium with and without siRNA-luc polyplexes. Figure 10b shows that the highest knockdown was observed when using siRNA polyplexes N/P 5 with 500 nM/well resulting in a knockdown of around 50% after 24 h of incubation. Considering the features of the mPEG-pDMAEMA polymer, its transfection activity is comparable to that of other polymers found in the literature. For instance, it is similar to the block copolymer studied by Lou et al. (approximately 34 kDa)^38^ and, in other cases, it even outperforms other polymers, such as when compared to polyethylenimine (25 kDa) and others.^42,77^ Furthermore, Lipofectamine 3000 was used as a positive control following manufacturing specifications (black bar), where a knockdown of ∼78% was observed. Lastly, incubation of cells with free siRNA (gray bar) did not result in luciferase knockdown, which confirms nanocarrier systems are needed for protection against degradation in the culture medium and the intracellular delivery of siRNA, which is in line with previous studies.^38,42,78,79^

## 4 Conclusions

In this study, stable pDNA and siRNA polyplexes were prepared using the mPEG-pDMAEMA diblock copolymer. Spheroids based on tumor cells only and combinations of tumor cells and fibroblasts were developed to mimic tumor ovarian nodules. The presented model is adaptable and reproducible for different ovarian cancer cells and showed to be a fast-screening tool for polyplex penetration, offering the possibility to test different conditions and find the most suitable formulation for preclinical applications (lowering the number of animals for in vivo studies). Using these spheroids, we proved that the size of the polyplexes is crucial for in vitro penetration. Tracking the penetration of small siRNA polyplexes into different types of homo- and heterospheroids, it was also confirmed that the presence of ECM produced by the fibroblasts retarded by not fully preventing the penetration of the polyplexes. Furthermore, studies involving 2D cocultured cells have highlighted the potential mechanism of siRNA polyplexes in penetrating the spheroids. This penetration likely occurs through a combination of passive diffusion, cellular uptake, and potential intercellular transport of polyplexes within the spheroids. To conclude, the results presented in this paper demonstrate for the first time that the designed siRNA polyplexes proved to be a potential candidate for ovarian cancer treatment capable to penetrate into in vitro 3D tumor nodules.

## Supporting Information Available

SiRNA sequences of siRNA-luc and siRNA-nc; ^1^H NMR spectrum of mPEG-pDMAEMA (PD); FCS time traces of free siRNA-Cy5 and siRNA polyplexes; polyplex siRNA concentration and loading capacity; SEM of pDNA polyplexes; agarose gel retardation assay pDNA and siRNA polyplexes; immunohistochemical staining of spheroids cryo-slices; brightfield images of A2780 and TOV112D spheroids, heterospheroids of A2780 and TOV112D cocultured with NIH3T3; polyplex association to SKOV3 homospheroids; and CLSM images of SKOV3-luc and NIH3T3 cells with siRNA-Cy5 polyplexes and free siRNA (PDF)
