# Supplementary material for: Effect of Polyplex Size on Penetration into Tumor Spheroids
Source: Mol Pharm. 2023 Oct 9;20(11):5515–31. doi: 10.1021/acs.molpharmaceut.3c00397 (PMC10630948; doi:10.1021/acs.molpharmaceut.3c00397)
Supplement: Supplementary file 1 — mp3c00397_si_001.pdf [file mp3c00397_si_001.pdf]

Supplementary Information

Effect of polyplex size on penetration into tumor spheroids

Cristina Casadidio[a,b], Jet E. M. Hartman[a], Bárbara S. Mesquita[a], Ragna Haegebaert[c], Katrien Remaut[c], Myriam Neumann[a], Jaimie Hak[a], Roberta Censi[b,e], Piera Di Martino[d,e], Wim E. Hennink[a] and Tina Vermonden[a,*]

[a] *Department of Pharmaceutical Sciences, division of Pharmaceutics, Utrecht Institute for Pharmaceutical Sciences (UIPS), Utrecht University 99, 3508 TB Utrecht, the Netherlands.*

[b] *School of Pharmacy, Drug Delivery Division, University of Camerino, CHiP Research Center, Via Madonna delle Carceri, 62032 Camerino (MC), Italy.*

[c] *Laboratory of General Biochemistry and Physical Pharmacy, Faculty of Pharmaceutical Sciences, Ghent University, 9000 Ghent, Belgium.*

[d] *Department of Pharmacy, 'G. D'Annunzio' University of Chieti and Pescara, Via dei Vestini 1, 66100 Chieti (CH), Italy.*

[e] *Recusol Srl, Via del Bastione 16, 62032 Camerino (MC), Italy*

Materials and Methods

Table S1. SiRNA sequences of both siRNA-luc and siRNA-nc used for cell viability & luciferase assay on 2D cell model.

Nucleic acid	Sense	Antisense
Luciferase siRNA (siRNA-luc)	5'-CUU-ACG-CUG-AGU-ACU-UCG-AdTdT-3'	5'-UCG-AAG-UAC-UCA-GCG-UAA-GdTdT-3'
Negative control siRNA (siRNA-nc)	5'-AUC-GUA-CGU-ACC-GUC-GUA-UdTdT-3'	5'-AUA-CGA-CGG-UAC-GUA-CGA-UdTdT-3'

Underlined bases indicate a 2'O-methyl modification. dT indicates a deoxyribonucleic acid base having phosphorothioate bond.

Polymer synthesis and characterization

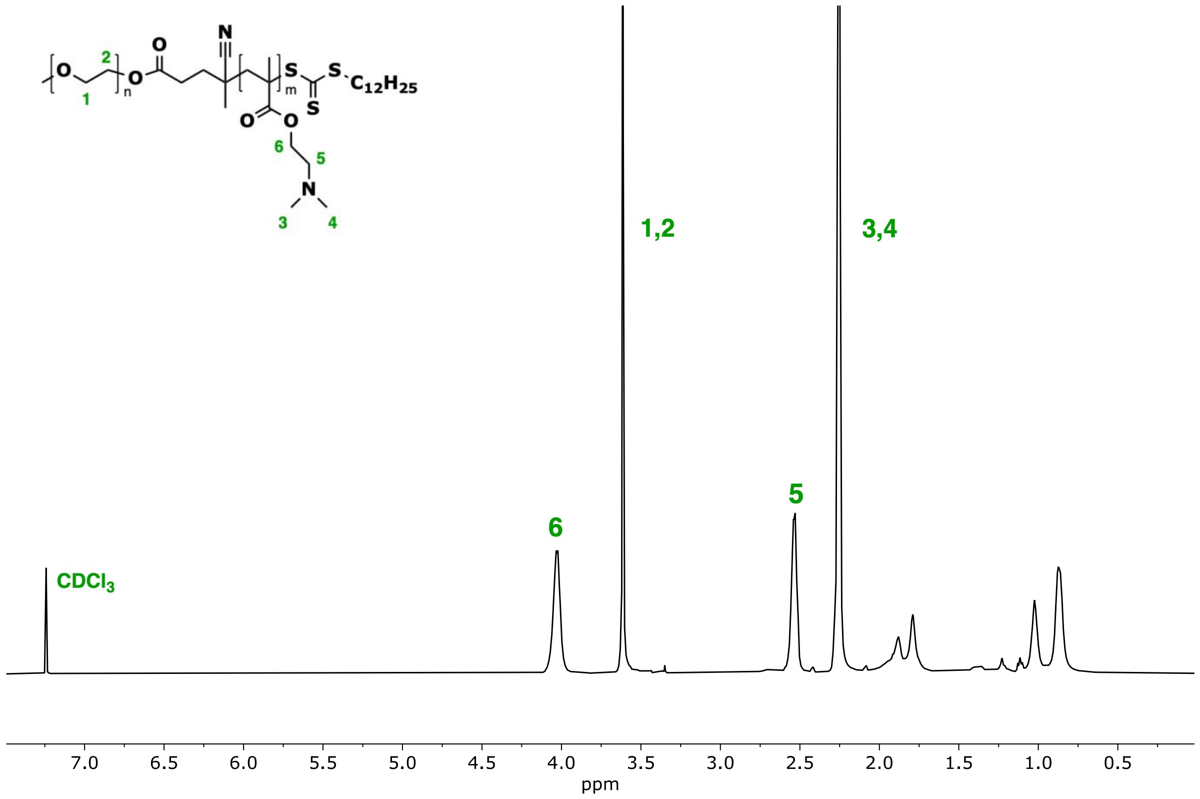

Figure S1. ^{1}H-NMR spectrum of mPEG-pDMAEMA (PD) diblock copolymer in $CDCl_3$.

Characterization of the polyplexes

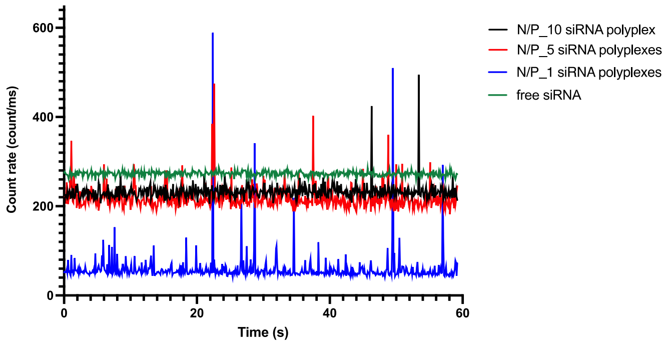

Figure S2. FCS time traces of free siRNA-Cy5 and siRNA polyplexes (N/P ratios of 1, 5 and 10) at RT.

Table S2. Polyplex siRNA concentration and loading capacity analyzed with fluorescence correlation spectroscopy (FCS). Free siRNA-Cy5 was included as control (n = 3, mean ± SD).

Formulations		Conc. (nM)	Conc. of free siRNA/Conc. of complex	Average number of siRNA molecules per complex	STD*
Free siRNA_1		46.0	n.a.**	n.a.	n.a.
Free siRNA_2		47.0	n.a.	n.a.	n.a.
Free siRNA_3		48.0	n.a.	n.a.	n.a.
siRNA polyplexes	N/P 1_1	0.8	62	63	27
	N/P 1_2	0.5	91		
	N/P 1_3	1.3	36		
	N/P 5_1	8.6	6	5	1
	N/P 5_2	10.0	5		
	N/P 5_3	9.3	5		
	N/P 10_1	15.0	3	3	1
	N/P 10_2	20.9	2		
	N/P 10_3	19.6	3		

*STD = standard deviation; **n.a. = not applicable

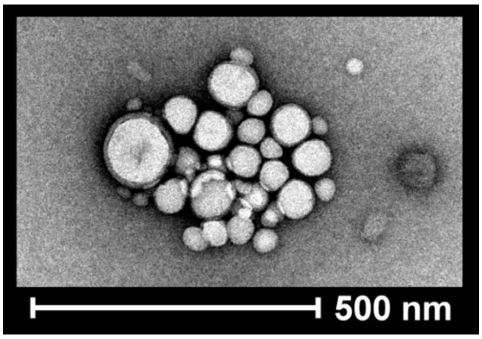

Figure S3. SEM image of pDNA polyplexes N/P ratio 5 prepared in 10 mM HEPES buffer.

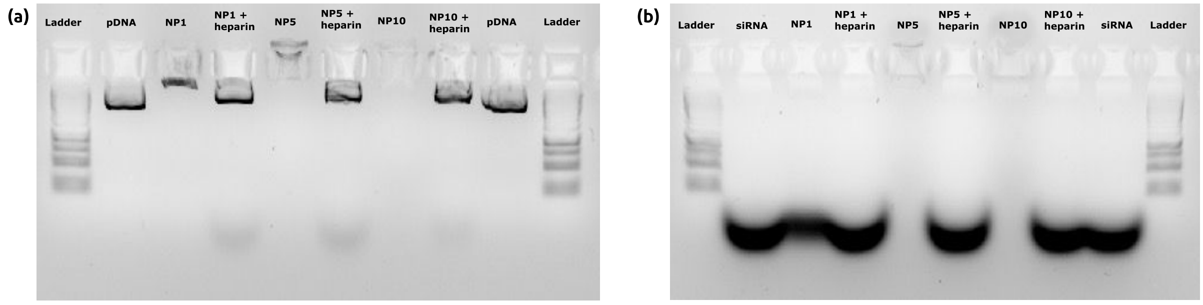

Figure S4. Agarose gel retardation assay to study the stability of (a) pDNA and (b) siRNA polyplexes with different N/P ratios ranging from 1 to 10 in HEPES buffer (10 mM, pH 7.4).

Histological and confocal characterization of SKOV3 spheroids

Method: SKOV3 homospheroids had been collected, embedded in Tissue Freezing Medium (Leica Biosystems, USA) and mounted in order to cut 8 μm-thick sections with Leica CM1520 cryostat. To first establish the presence of spheroids in specific sections, sections were fixed with acetone for 10 minutes, washed 5 minutes with PBS three times and stained with Hematoxylin & Eosin Y (H&E) for 60 and 45 seconds respectively, with 3 washing steps of water in between the staining. Then, sections were dried with ethanol 96 and 100% for 60 and 30 seconds respectively, in order to mount them with DPX and a cover glass. Slides were visualized using a 40x objective to determine which sections contain spheroids so further analyzes could be done on the consecutive sections. These were then fixed and washed the same way, but stained with Hoechst 33342 for 20 minutes instead of H&E. Thereafter, the sections were washed with PBS 2 times and mounted with fluorsave, before imaging in a 37 °C enclosed incubator with Leica TCS SP8 SMD scanning unit (Leica Microsystems, Wetzlar, Germany) and a Leica DMI6000 inverted microscope (objective 10x). CLSM images were recorded at excitation wavelengths of 405 and 488 nm for Hoechst 33342 and CellTracker™ Green CMFDA.

Results: The mounting procedure of SKOV3 spheroids can be found as the white solid within the purple mounting medium (Fig. S4a). When the spheroids were cut into several cryo-slices, Hematoxylin & Eosin Y (H&E) staining confirmed the presence of spheroid and its morphology, proving that the spheroids developed are fully packed with cells (even in the core) without a dip in their shape (Fig. S4b). Results were confirmed by CLSM imaging, where is possible to distinguish the ovarian cancer cell lines (green) with their nuclei (blue) (Fig. S4c).

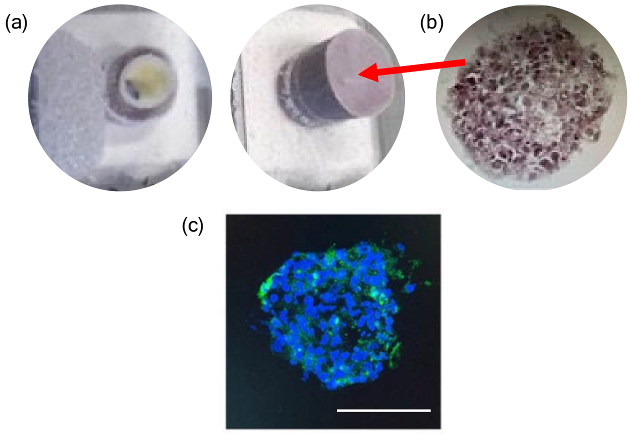

Figure S5. Immunohistochemical staining of spheroids cryo-slices. (a) Images show the mounting technique used for freezing and thereafter cutting planes of spheroids. (b) Microscopic images of a successful H&E staining, showing cell nuclei and extracellular matrix of a spheroids cryo-slice. (c) CLSM images of the spheroids cryo-slice, showing the cells (green) with their cell nuclei (blue). Scale bar = 100 μm.

Characterization of the spheroids

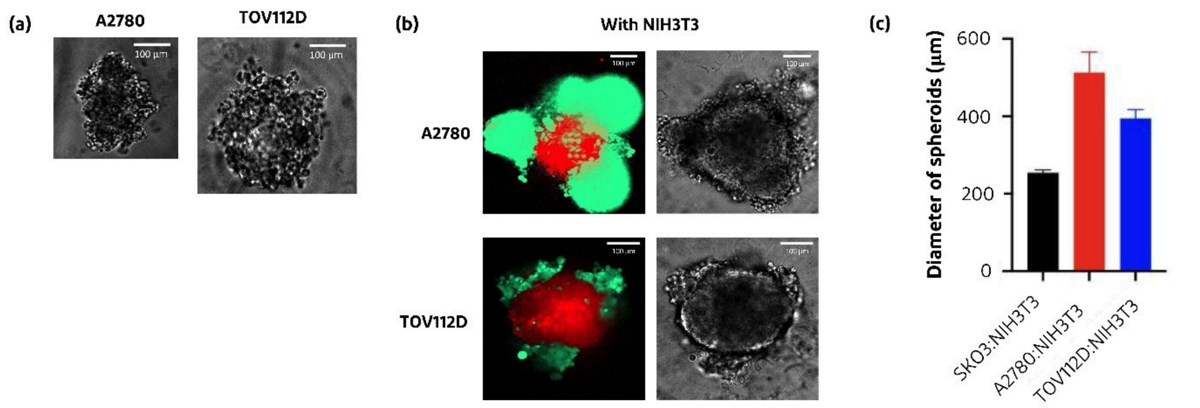

Figure S6. (a) Brightfield images of A2780 and TOV112D spheroids resulting in a total cell density of 100 cells/well. (b) Formation of heterospheroids, composed by different ovarian carcinoma cell lines (A2780 and TOV112D) co-cultured with NIH3T3 (cell ratio was 1:1), analyzed by CLSM. Ovarian cancer cells stained with green cell tracker while NIH3T3 fibroblasts are stained with a red cell tracker resulting in a total cell density of 400 cells/well. (c) Calculated diameters of different heterospheroids (ratio 1:1).

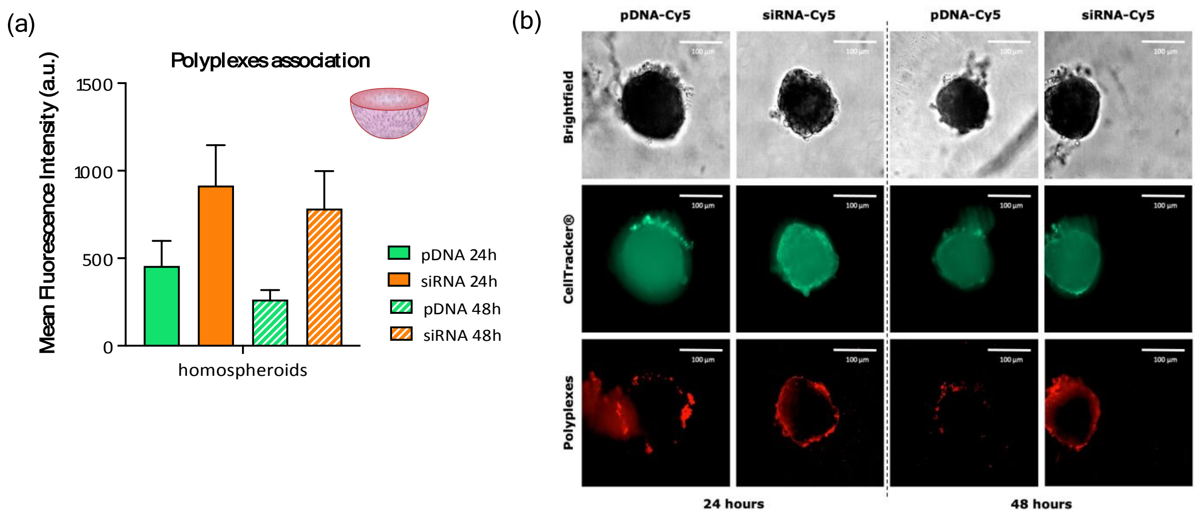

Figure S7. Association of polyplexes into SKOV3 homospheroids after 24 and 48 hours of incubation. (a) Total mean fluorescent intensity (MFI) of the pDNA-Cy5 vs siRNA-Cy5 polyplexes association within the SKOV3 homospheroids after 24 and 48 hours of incubation at 37 °C (n= 3-6), calculated as described in section 2.9. *p<0.05, **p < 0.01. (b) CLSM penetration images of pDNA and siRNA polyplexes into SKOV3 homospheroids. Incubation times of both 24 (left) and 48 hours (right) at 37°C were investigated. Scale bar = 100 μm.

Uptake studies on 2D cell layout

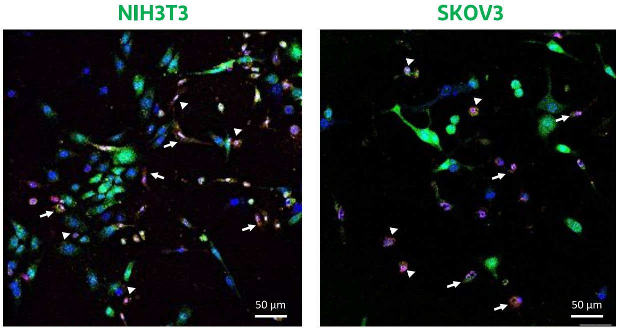

Figure S8. CLSM images of monoculture SKOV3-luc and NIH3T3 cells after 24 hours of incubation with siRNA-Cy5 polyplexes at 37 °C in full medium. Cell Tracker corresponds to the green color (CellTracker™ Green CMFDA Dye), cell nuclei to blue (Hoechst 33342) and siRNA to red (siRNA-Cy5).

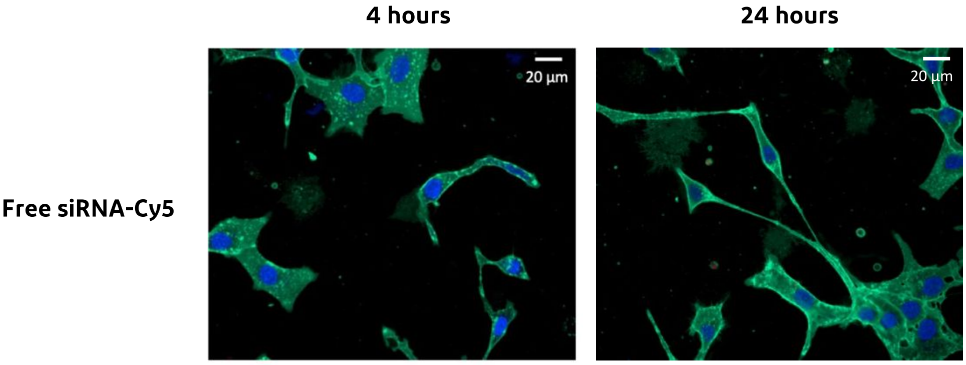

Figure S9. CLSM images of SKOV3-luc cells after 4 and 24 hours of incubation with free siRNA at 37 °C in full medium. Cell membranes correspond to the green color (Wheat Germ Agglutinin-Alexa Fluor 488), cell nuclei to blue (Hoechst 33342) and siRNA to red (siRNA-Cy5).
